# Efficient activation of hundreds of LTR12C elements reveals *cis*-regulatory function determined by distinct epigenetic mechanisms

**DOI:** 10.1093/nar/gkae498

**Published:** 2024-06-14

**Authors:** Hitoshi Ohtani, Minmin Liu, Gangning Liang, H Josh Jang, Peter A Jones

**Affiliations:** Department of Epigenetics, Van Andel Research Institute, Grand Rapids, MI 49503, USA; Department of Animal Sciences, Graduate School of Bioagricultural Sciences, Nagoya University, Chikusa-ku, Nagoya, Aichi 464-8601, Japan; Department of Epigenetics, Van Andel Research Institute, Grand Rapids, MI 49503, USA; Department of Urology, Keck School of Medicine, University of Southern California, Los Angeles, CA 90089, USA; Department of Epigenetics, Van Andel Research Institute, Grand Rapids, MI 49503, USA; Department of Epigenetics, Van Andel Research Institute, Grand Rapids, MI 49503, USA

## Abstract

Long terminal repeats (LTRs), which often contain promoter and enhancer sequences of intact endogenous retroviruses (ERVs), are known to be co-opted as *cis-*regulatory elements for fine-tuning host-coding gene expression. Since LTRs are mainly silenced by the deposition of repressive epigenetic marks, substantial activation of LTRs has been found in human cells after treatment with epigenetic inhibitors. Although the LTR12C family makes up the majority of ERVs activated by epigenetic inhibitors, how these epigenetically and transcriptionally activated LTR12C elements can regulate the host-coding gene expression remains unclear due to genome-wide alteration of transcriptional changes after epigenetic inhibitor treatments. Here, we specifically transactivated >600 LTR12C elements by using single guide RNA-based dCas9-SunTag-VP64, a site-specific targeting CRISPR activation (CRISPRa) system, with minimal off-target events. Interestingly, most of the transactivated LTR12C elements acquired the H3K27ac-marked enhancer feature, while only 20% were co-marked with promoter-associated H3K4me3 modifications. The enrichment of the H3K4me3 signal was intricately associated with downstream regions of LTR12C, such as internal regions of intact ERV9 or other types of retrotransposons. Here, we leverage an optimized CRISPRa system to identify two distinct epigenetic signatures that define LTR12C transcriptional activation, which modulate the expression of proximal protein-coding genes.

## Graphical Abstract

**Graphical Abstract:**
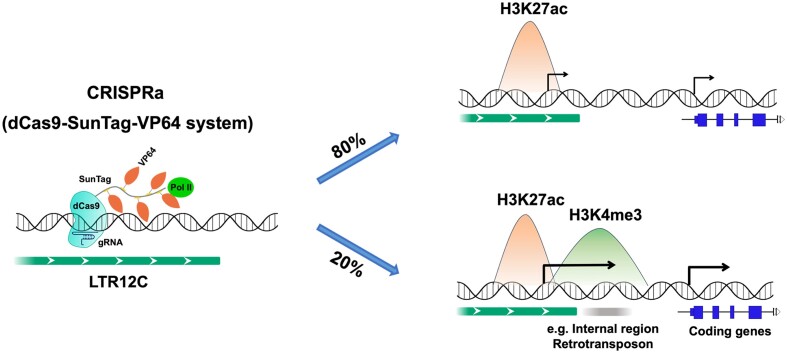

## Introduction

Human endogenous retroviruses (ERVs) make up >8% of the host human genome by their repetitive nature (1). While ERVs are no longer actively transposing in the human genome, recent reports suggest that resurrected ERVs can play important roles in fine-tuning host gene expression as *cis*-regulatory elements, such as by acting as promoters or enhancers (2–7). Since the majority of ERVs are silenced by epigenetic modifications (3,6,7), the *cis*-regulatory element activity has been reported extensively in cells treated with inhibitors of epigenetic processes, such as DNA methyltransferases, histone deacetylases and histone methyltransferases (3,8–10). While the properties of ERVs have been difficult to analyze due to their repetitive sequence nature (6,11), recently developed sequencing technologies coupled with new computational strategies now allow for the mapping of specific repetitive elements (12,13). We and other groups have conducted a detailed dissection of ERV expression in human cells following treatments with epigenetic inhibitors, including the DNA methylation inhibitor 5-aza-2′-deoxycytidine (5-aza-CdR), histone methyltransferase G9a inhibitor or histone deacetylase inhibitor (HDACi) (8,14–18). It is noteworthy that although ∼400 ERV families are categorized in RepeatMasker (http://www.repeatmasker.org), the LTR12C subfamily, which consists of solitary long terminal repeats (LTRs) derived from promoter and enhancer regions of ERV9, makes up a large fraction of induced ERV families after treatment with epigenetic inhibitors (8,15,17). While LTR12C has been well documented to provide *cis-*regulatory function (8,19,20), to what degree activated LTR12C regulates the host gene expression is still not fully elucidated due to the broad transcriptional changes and global alteration of the epigenetic landscape engendered by the inhibitor treatments.

On the other hand, recent developments of site-specific targeting technology CRISPR activation (CRISPRa) have been instrumental in understanding the action mechanisms of *cis*-regulatory elements, especially promoters and enhancers, on gene expression (21). dCas9-VP64, which consists of catalytically dead Cas9 (dCas9) fused with the transcriptional activator VP64, has been used as one representative CRISPRa system (22,23). In addition, Hilton *et al.* showed that the dCas9-p300-based CRISPRa system consisting of dCas9 fused with the histone acetyltransferase domain of E1A-associated protein p300 had an increased capacity for transactivation targeting protein-coding genes relative to dCas9-VP64 (24). It is known that not only *cis*-regulatory elements for coding genes but also some types of ERVs can also be targeted by these CRISPRa systems (24–26). Furthermore, Morita *et al.* reported that the epigenome editing dCas9-SunTag-TET1 system, consisting of dCas9 fused with SunTags separated by 22-amino-acid linkers that recruit multiple copies of ten–eleven 1 hydroxylase (TET1), was highly effective in manipulating DNA methylation states compared to the canonical system composed of SunTags separated by 5-amino-acid linkers (27,28). Therefore, future implementation of a CRISPRa system containing SunTags separated by 22-amino-acid linkers may improve the activation efficiency of the *cis*-regulatory elements.

Here, we precisely transactivated LTR12C using two developed CRISPRa systems, dCas9-SunTag-VP64 and dCas9-SunTag-p300, in human cells. Contrary to expectation, dCas9-SunTag-VP64 had an increased capacity for transactivation of LTR12C relative to dCas9-SunTag-p300. While most of the activated LTR12C elements were characterized by an H3K27ac-marked enhancer signature, ∼20% of them were characterized by both an H3K4me3-marked promoter and an H3K27ac-marked enhancer signature. These distinct regulatory signatures have implications for fine-tuning downstream host gene expression. In conclusion, we leveraged the power of precise CRISPRa technology to show that LTR12C elements may have acquired locus-dependent regulatory element activities by their colonization during host human genome evolution.

## Materials and methods

### Cell lines and 5-aza-CdR treatment

HEK293T (human embryonic kidney), HCT116 (human colorectal carcinoma) and MCF7 (human breast carcinoma) cells were obtained from the American Type Culture Collection. A2780 (human ovarian carcinoma) cells were a gift from Dr Stephen B. Baylin’s laboratory. HEK293T and MCF7 cells were maintained in Dulbecco’s modified Eagle medium with 10% fetal bovine serum (FBS) and antibiotics (penicillin and streptomycin). HCT116 cells were maintained in McCoy’s 5A medium with 10% FBS and antibiotics. A2780 cells were maintained in RPMI 1640 medium with 10% FBS and antibiotics. HEK293T cells were treated with phosphate-buffered saline (PBS; control) or 300 nM 5-aza-CdR for 24 h. The cells were harvested at 5 days after treatment.

### Plasmid constructs

pPlatTET-gRNA2 (Addgene #82559) plasmid served as a source vector to create dCas9-SunTag-VP64 and dCas9-SunTag-p300. The VP64 sequence was amplified from pcDNA-dCas9-VP64 plasmid (Addgene #47107) by polymerase chain reaction (PCR). The p300 sequence corresponding to amino acids 1048–1664 was amplified from pcDNA-dCas9-p300 core by PCR (Addgene #61357). The fragments were inserted into pPlatTET-gRNA2 by Gibson assembly (29) after cutting out the TET1 sequence. The guide RNAs (gRNAs) targeting the promoter regions of *RHOXF2B*, *IL1RN* and *OCT4* or nontargeting gRNAs were designed based on previous reports (24,27). The gRNAs targeting LTR12C were designed in consensus sequence of LTR12C elements in RepeatMasker. Plasmid constructs were created by incorporating the gRNA fragments into linearized dCas9-SunTag-VP64 or dCas9-SunTag-p300 plasmid based on Gibson assembly. The gRNA sequences are listed in Supplementary Table S3.

### Plasmid transfection

Transfections were performed by Lipofectamine LTX Reagent (Thermo Fisher Scientific) with 500 ng plasmid constructs into HEK293T, HCT116, MCF7 or A2780 cells as per the manufacturer’s instructions. The cells were harvested after 3 days. Subsequently, fluorescence-activated cell sorting (FACS) was performed at the Van Andel Research Institute Flow Cytometry Core to isolate GFP-positive HCT116, MCF7 or A2780 cells to separate out the cells that had insufficient transfection efficiency. Since sufficient number of GFP-positive cells were obtained as described in the Lipofectamine LTX Reagent protocol (>80% transfection efficiency), we did not conduct FACS to minimize potential artifacts introduced by flow sorting in HEK293T cells.

### RNA-seq library construction and analysis

Total RNA from cell lines after CRISPRa was purified using a Direct-zol RNA MiniPrep Kit (Zymo Research). Complementary DNA (cDNA) libraries were prepared using RNA HyperPrep Kits with RiboErase (KAPA Biosystems) according to the manufacturer’s instructions, and were sequenced as single-end 75 bases on a NextSeq 500 instrument (Illumina). Sequencing reads were aligned against the human GRCh37/hg19 reference genome to report consistent results with our previous study (15) by using HISAT2. The definitions of ERV elements were based on RepeatMasker (http://www.repeatmasker.org). RPKM (reads per kilobase per million mapped reads) values were calculated using uniquely mapped reads obtained from featureCounts. The raw and processed datasets from this study have been submitted to the NCBI Gene Expression Omnibus (GEO; http://www.ncbi.nlm.nih.gov/geo/) under accession number GSE243551. Source code was deposited in Figshare (doi: 10.6084/m9.figshare.24906660).

### RT-qPCR

cDNA synthesis was performed with an iScript cDNA Synthesis Kit (Bio-Rad) after DNA digestion by TURBO DNase (Thermo Fisher Scientific) according to the manufacturer’s instructions. Quantitative PCR (qPCR) reactions were carried out in a CFX96 Real-Time PCR Detection System (Bio-Rad) with a KAPA SYBR FAST qPCR Kit (KAPA Biosystems). The conditions of qPCR reactions were as follows: 3 min at 95°C followed by 40 cycles of 10 s at 95°C, 20 s at 60°C and 20 s at 72°C. Primers are listed in Supplementary Table S4.

### ChIP-seq library construction and analysis

Transfected cells were cross-linked with 1% formaldehyde for 10 min and the cross-linking was neutralized with 125 mM glycine for 5 min at room temperature. Cell pellets were lysed in 1% lysis buffer [50 mM HEPES–KOH, pH 7.4, 150 mM sodium chloride, 1 mM ethylenediaminetetraacetic acid (EDTA), 1% Triton X-100, 0.1% sodium deoxycholate, 0.1% sodium dodecyl sulfate, 1× cOmplete Mini EDTA-free Protease Inhibitor Cocktail]. The cross-linked chromatin was sheared into ∼300 base pairs using Covaris S2 ultrasonicators. The fragmented chromatin was incubated with anti-HA-tag antibody (Cell Signaling, C29F4), anti-H3K4me3 antibody (Diagenode, C42D8) or anti-H3K27ac antibody (Active Motif, MABI 0309) immobilized on Dynabeads Protein G overnight at 4°C. The beads were washed with 1% lysis buffer on a magnetic rack followed with TE (10 mM Tris–HCl, pH 8.0, 1 mM EDTA). After reversing cross-linking for 2 h at 65°C, the samples were treated with RNase A/T1 for 30 min at 37°C followed by Proteinase K digestion for 1 h at 65°C, and then DNA was recovered. Sequencing libraries were prepared using a TruSeq ChIP Library Prep Kit (Illumina) and sequenced as single-end 75 bases on a NextSeq 500 instrument (Illumina) at the Van Andel Research Institute Genomics Core. Sequencing reads were aligned against the human GRCh37/hg19 reference genome to report consistent results with our previous study (15) by using Bowtie2. Sequencing data are available through GEO (accession number GSE243551). Source code was deposited in Figshare (doi: 10.6084/m9.figshare.24906660).

### Whole genome bisulfite sequencing

Genomic DNA from HEK293T after CRISPRa was purified using DNAzol (Invitrogen) according to the manufacturer’s instructions. Bisulfite conversion and library preparation were performed by the Van Andel Research Institute Genomics Core. Paired-end 150 bp sequencing was also conducted by the Genomics Core on the Illumina HiSeq platform. In addition, we obtained whole genome bisulfite sequencing data of HEK293T cells from a published work (30) to explore basal DNAme levels of LTR12C elements (Supplementary Figure S2A). The sequencing reads were aligned against the human GRCh37/hg19 reference genome by using BSMAP (31). The DNA methylation levels at each CpG site were calculated as the ratio of methylated CpGs and all CpGs in uniquely mapped reads. Sequencing data are available through GEO (accession number GSE243551). Source code was deposited in Figshare (doi: 10.6084/m9.figshare.24906660).

### Prediction of TFBSs

We utilized the MEME Suite (32) to perform sequence motif discovery in the genomic sequences within 500 bp downstream region of co-marked LTR12Cs (*n* = 130) or single-marked LTR12Cs (*n* = 439). We showed two significant and enriched motifs (*E*-value < 1 × 10^230^) within the downstream regions of co-marked LTR12Cs in Supplementary Figure S9. Subsequently, the predictions of TFBSs on the two motifs were conducted by TomTom (33). We filtered any transcription factor motifs that were detected in the downstream regions of single-marked LTR12Cs to provide unique motif candidates solely found in the downstream regions of co-marked LTR12C. We further filtered out motif candidates from transcription factors showing low expression level in HEK293T cells (RPKM < 1).

## Results

### LTR12C is most abundantly activated in four distinct human cell lines after treatment with DNA methylation inhibitor 5-aza-CdR

**Figure 1.**
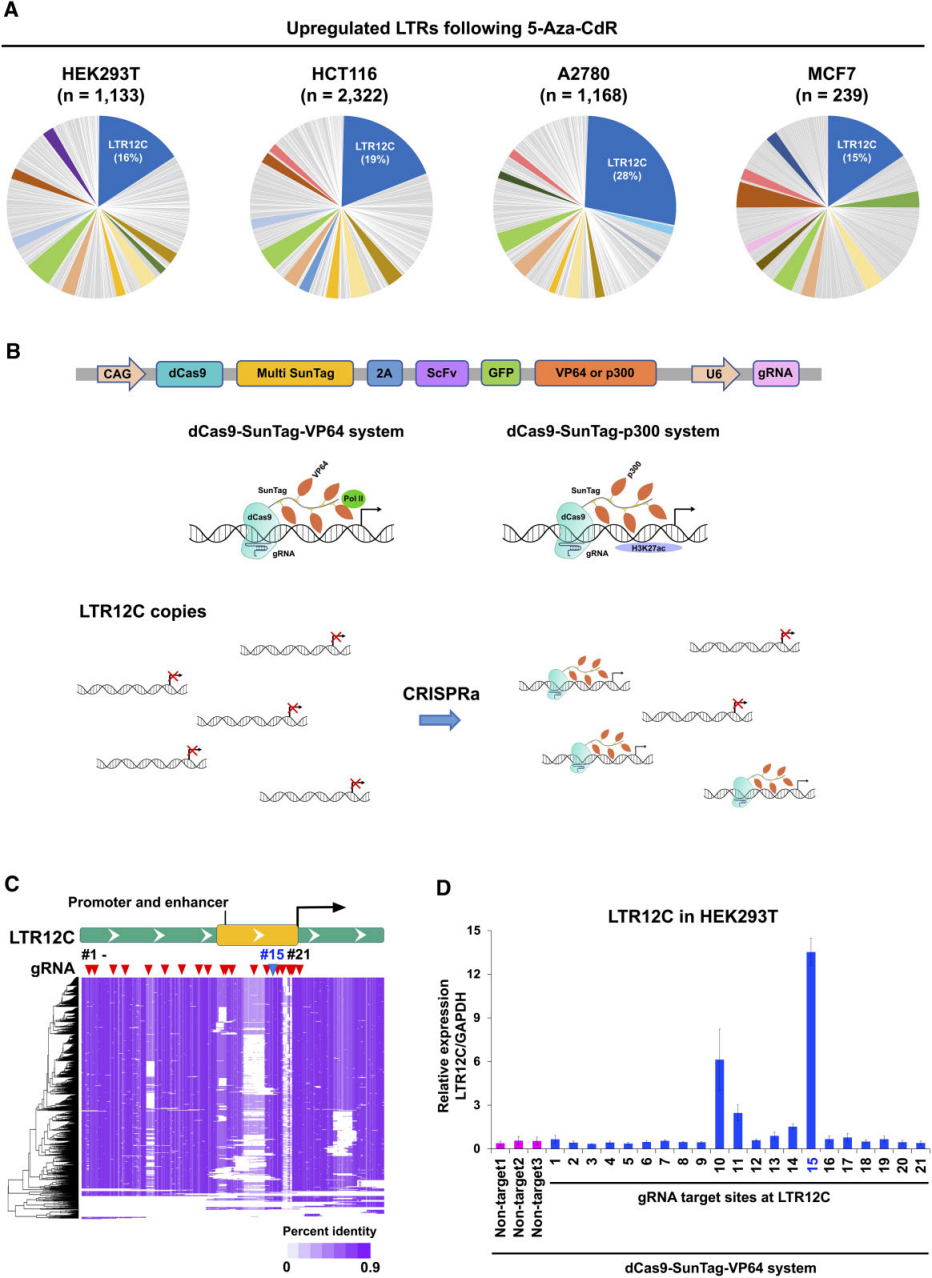
The dCas9-SunTag-based CRISPRa systems induce LTR12C expression from proximal promoter regions. (**A**) Percentage of upregulated LTR families in human cell lines following 5-aza-CdR treatment. The four cell lines were treated with PBS (control) or 300 nM 5-aza-CdR for 24 h and harvested at 5 days after the treatment. The upregulated LTR copies [fold change (FC) >2 by comparison with PBS-treated human cell lines] are categorized into each LTR family. The top 10 most highly abundant LTR families are highlighted with distinct colors. (**B**) Schematics of dCas9 constructs and fusion proteins. The constructs contain dCas9, SunTags separated by 22-amino-acid linkers (Multi SunTag), 2A self-cleaving peptide (2A), single-chain variable fragment (ScFv), green fluorescence protein (GFP), VP64/p300 and gRNA. The fusion proteins with single gRNA are inferred to transactivate multiple copies of LTR12C. (**C**) A sequence alignment of LTR12C elements deposited in the RepeatMasker database. Each row represents one LTR12C element. Heatmap indicates percent identity (0 to 90%); blank indicates gaps. Arrowhead indicates gRNA positions. Promoter and enhancer region is defined as 400 bp upstream region from TSS. (**D**) Relative expression of LTR12C based on reverse transcription qPCR (RT-qPCR) after transfection of the dCas9-SunTag-VP64 construct with represented gRNAs in HEK293T cells. Error bars represent standard error of the mean (SEM) from three independent biological replicates.

Recent genome-wide studies of repetitive elements from our and other groups reported a potent induction of LTR12C expression in human cells following treatment with epigenetic inhibitors (8,15,17). The activated LTR12C elements were also likely co-opted for their *cis-*regulatory potential, such as promoter or enhancer activity, to regulate nearby host-coding gene expression (8). To confirm the promiscuous activation of LTR12C across various types of human cell lines, we performed comparative transcriptomic analyses of induced LTRs by incorporating published RNA sequencing (RNA-seq) datasets of colorectal carcinoma cells (HCT116), breast carcinoma cells (MCF7) and ovarian carcinoma cells (A2780) treated with 5-aza-CdR (17) and generated additional RNA-seq libraries from embryonic kidney cells (HEK293T) for this study. Although ∼400 LTR families are recognized in RepeatMasker, the largest fraction of the activated LTR elements was derived from the specific LTR12C family across these cell lines, as expected (Figure 1A). The results imply the existence of potential important and conserved roles of LTR12C as *cis-*regulatory elements for fine-tuning host-coding gene expression.

### LTR12C was substantially more transactivated by dCas9-SunTag-VP64 than by dCas9-SunTag-p300

To decouple LTR12C-specific activation from the global epigenetic alterations exerted by epigenetic inhibitor treatment, we engineered dCas9 with SunTags separated by 22-amino-acid linkers that can recruit the VP64 transactivator domain or p300 histone acetyltransferase domain (Figure 1B). Since LTR12C is a family of repetitive elements, we designed gRNAs that target evolutionarily conserved sequences in LTR12C copies to simultaneously target numerous copies of LTR12C with a single gRNA (Figure 1B and C). To select highly efficient gRNA for the transactivation, we assessed on-target activities of 21 distinct gRNAs, which were designed around the transcription start site (TSS) and upstream regions including enhancer and promoter sequences of LTR12C (Figure 1C and Supplementary Figure S1). gRNA #15, which is located at ∼100 bases upstream from the TSS and is flanked by pertinent TFBSs (8,19,20,34) (Supplementary Figure S1), showed the highest transactivation with dCas9-SunTag-VP64-based CRISPRa (Figure 1C and D). The second highest transactivation was achieved by gRNA #10 suggesting that the region between gRNAs #10 and #15 contains certain sequence features that dictate LTR12C transcriptional activity. We focused on pursuing gRNA #15 for downstream analysis as the targeting sequence of gRNA #15 is perfectly conserved in 24% of LTR12C copies and 64% of LTR12Cs if we allow one mismatch in the gRNA target sequence.

**Figure 2.**
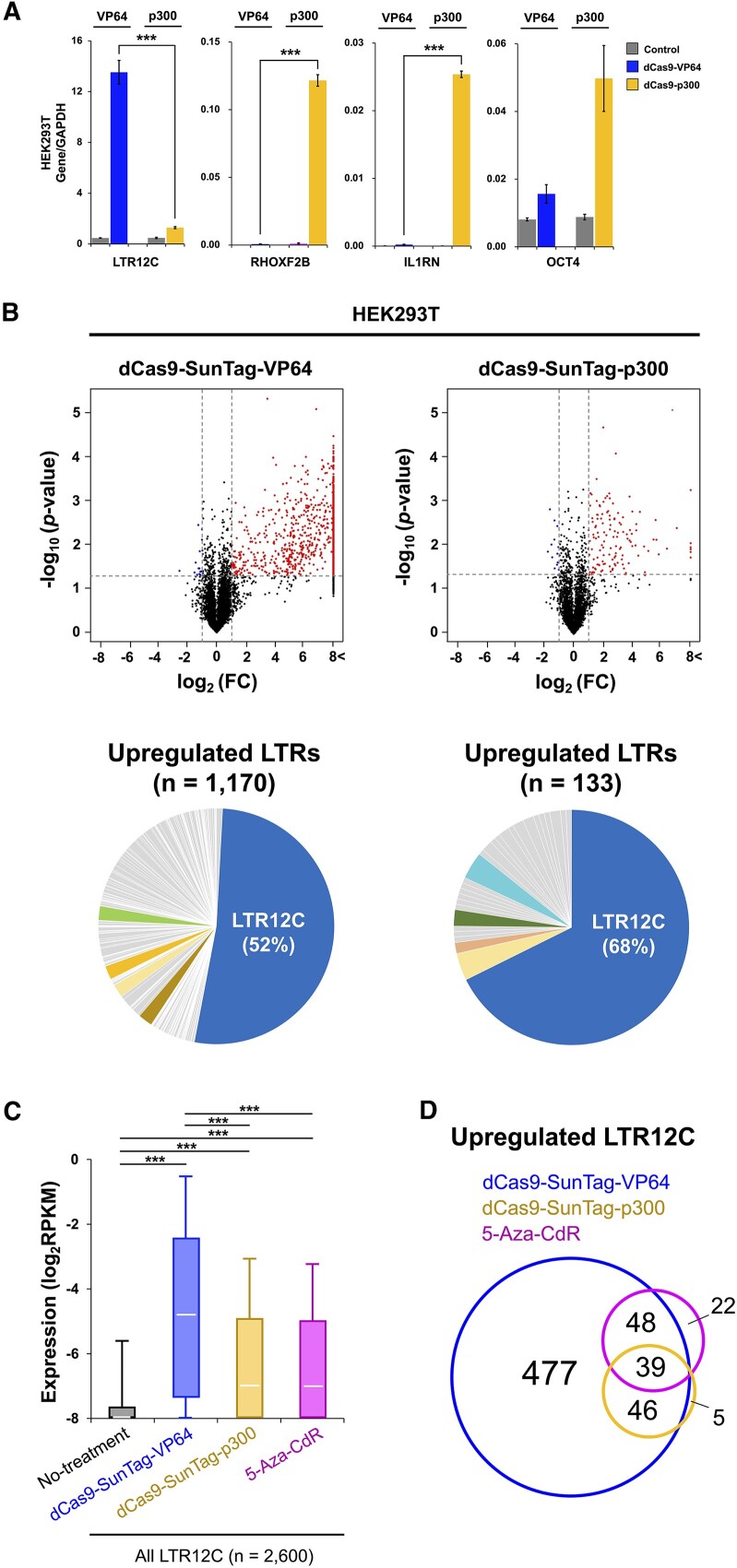
The transactivation capacity of the dCas9-SunTag-VP64 system for LTR12C is higher than that of the dCas9-SunTag-p300 system. (**A**) Relative RNA expression of LTR12C and selected coding genes for *RHOXF2B*,*IL1RN* and *OCT4* following represented CRISPRa as determined by RT-qPCR in HEK293T cells. Primers of LTR12C were designed at multiple loci. Error bars represent SEM from three independent biological replicates. *P*-values were calculated using the two-tailed Student’s *t*-test: ***P < 0.001. (**B**) The volcano plots show expression changes of LTR copies (not only LTR12C) after transfection of represented constructs with LTR12C-targeting gRNA in HEK293T cells. The log_2_FC values and −log_10_(*P*-value) were calculated by comparison with expression of LTR copies in cells transfected with constructs expressing nontargeting gRNA. Dashed lines are the threshold of the two-tailed Wilcoxon signed-rank test *P*-value <0.05 (horizontal) or FC > 2 (vertical). The red (upper-right area) and blue (upper-left area) points represent upregulated and downregulated LTR copies with statistical significance, respectively. The pie charts show percentage of the upregulated LTR copies in each LTR family. The top five most highly abundant LTR families are highlighted with distinct colors. (**C**) The distribution of expression level (RPKM) of LTR12C in the represented groups in HEK293T cells. ‘All LTR12C’ refers to LTR12C copies that were deposited in RepeatMasker. Each box represents the data between the 25th and 75th quartiles. The whiskers are drawn down to the 10th percentile and up to the 90th percentile. White bars indicate median values. Adjusted *P*-values were calculated using Mann–Whitney *U* test and Holm’s method: ***P < 0.001. (**D**) LTR12C copies overlapping among upregulated LTR12C by dCas9-SunTag-VP64, dCas9-SunTag-p300 and 5-aza-CdR in HEK293T cells.

Since an earlier study reported that the transactivation capacity of the dCas9-p300 system for protein-coding genes was significantly higher than that of the dCas9-VP64 system (24), we also attempted to transactivate LTR12C by our dCas9-SunTag-p300 system. Contrary to expectation, dCas9-SunTag-p300 induced a significantly lower expression level of LTR12C than dCas9-SunTag-VP64 (Figure 2A). To verify the general superiority of dCas9-p300 in transactivation of protein-coding genes, we targeted *RHOXF2B*, *IL1RN* and *OCT4* genes by using our dCas9-SunTag-p300 system (Figure 2A) (24). Indeed, dCas9-SunTag-p300 induced stronger transcriptional activation across these protein-coding genes (Figure 2A). This suggests that the differential activation of LTR12C elements might be influenced by specific sequence or epigenetic contexts. To explore the underlying mechanisms responsible for the difference, we performed transcriptome profiling of LTR12C copies by total RNA-seq on HEK293T cells after dCas9-SunTag-VP64- or dCas9-SunTag-p300-based CRISPRa. The dCas9-SunTag-VP64 transcriptionally activated 1,170 LTR copies, of which 52% were LTR12C (Figure 2B). Although 68% of total activated LTR copies are LTR12C by dCas9-SunTag-p300, the total number of LTR copies was much lower (*n* = 133) compared to LTRs (*n* = 1,170) activated by dCas9-SunTag-VP64 (Figure 2B). Furthermore, the expression level of LTR12C copies that were transactivated by dCas9-SunTag-VP64 was much greater in magnitude than those that were transactivated by dCas9-SunTag-p300 or upregulated by 5-aza-CdR treatment (Figure 2C). We next compared the LTR12C activated by dCas9-SunTag-VP64 to the LTR12C activated by dCas9-SunTag-p300 and found that there was a large overlap (Figure 2D). Since histone acetyltransferase p300 regulates gene expression by predominately acting through H3K27ac modification at promoter and enhancer regions, we performed comparative analyses among the groups based on basal (state before CRISPRa) epigenetic modifications or gene expression level of LTR12C elements to explore interpretable features that contribute to transactivation efficiency of dCas9-SunTag-VP64 or dCas9-SunTag-p300. Whereas the two LTR12C groups displayed no distinguishable differences in basal DNA methylation and H3K27ac levels (Supplementary Figure S2A and B), a significant difference in basal LTR12C expression was found (Supplementary Figure S2C). These results indicate that dCas9-SunTag-p300 mainly induced LTR12C copies that were already transcriptionally permissive, while dCas9-SunTag-VP64 has a lower target preference on LTR12C copies. Of note, several LTR12C copies induced by 5-aza-CdR treatment were also weakly transcribing before the treatment, which largely overlapped with dCas9-SunTag-VP64-targeted LTR12C copies (Figure 2D and Supplementary Figure S2C).

We also performed CRISPRa by the dCas9-SunTag-VP64 system on three different human cancer cell lines (HCT116, A2780 and MCF7) to verify that the LTR12C activation potential was not a cell-type-specific phenomenon. Indeed, we observed robust transactivation of LTR12C by dCas9-SunTag-VP64, where 98.7% (602/610) or 79.0% (482/610) of transactivated LTR12C elements in HEK293T were also detected in at least one or all three cancer cell lines, respectively (Supplementary Figure S3A and B). Of note, we detected higher number of transactivated LTR12C in the cancer cell lines compared to the ‘normal’ HEK293T cells, which could be attributed to increased promiscuity of LTR12C elements shaped by global DNA hypomethylation, which is a hallmark of cancer cells. Ultimately, we show here that dCas9-SunTag-VP64 can confer robust transactivation for potential transcriptionally competent LTR12C elements. However, further study is needed to elucidate the unknown molecular mechanisms for the superiority of dCas9-SunTag-VP64 in transactivation of LTR12C.

### dCas9-SunTag-VP64 exhibited minimal off-target activity

**Figure 3.**
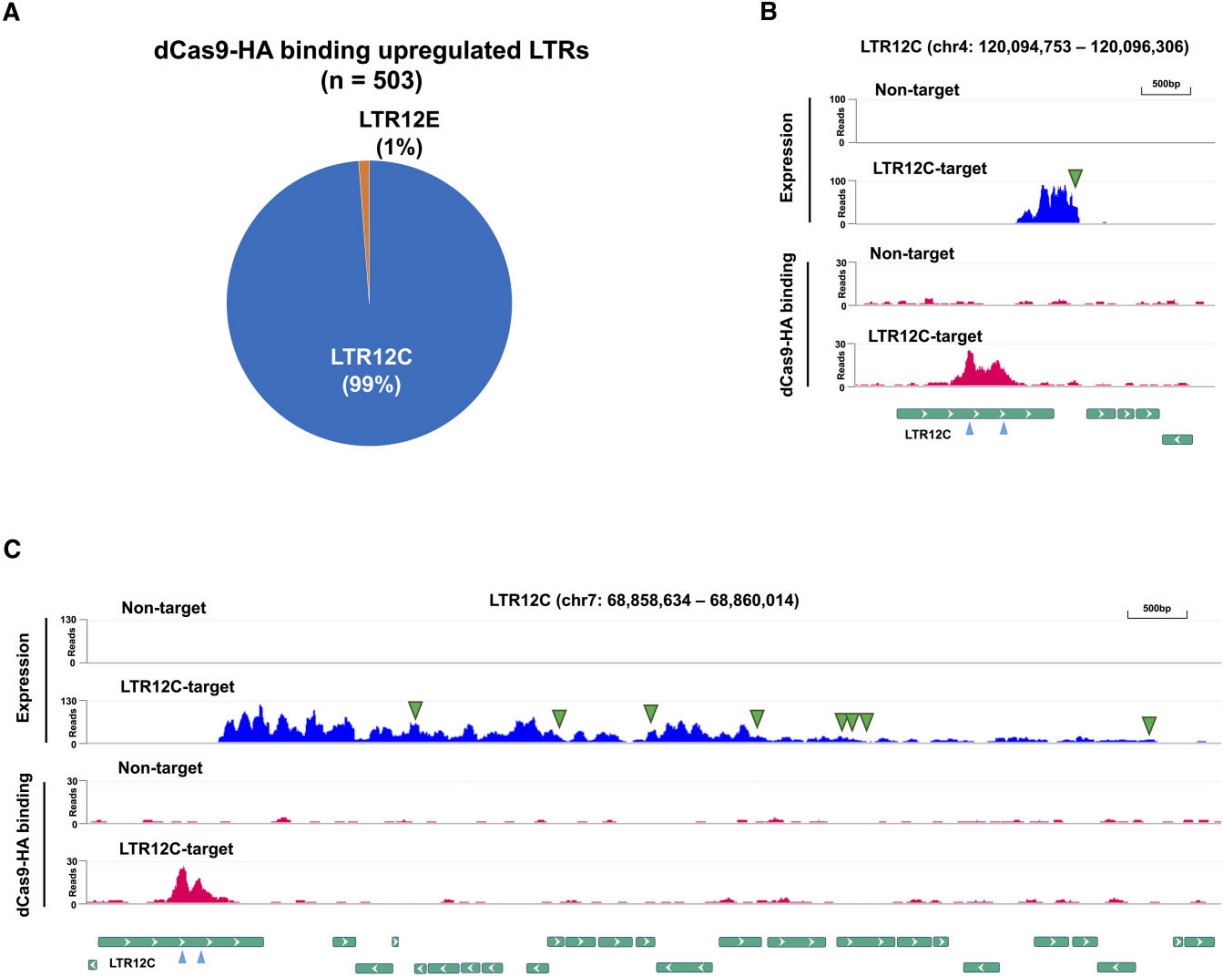
The off-target events by single gRNA-based dCas9-SunTag-VP64 were limited. (**A**) Percentage of dCas9-HA binding LTR families represented among the LTRs upregulated after dCas9-SunTag-VP64-based CRISPRa in HEK293T cells, as detected in Figure 2B, left panel. (**B**) Representative genomic regions showing LTR12C-specific transactivation in HEK293T cells. The track views represent RNA expression based on RNA-seq (upper) and dCas9-HA binding sites based on ChIP-seq (lower). Green (bottom) bars indicate genomic position of retrotransposons with their strand orientation. Arrowhead (blue) on the retrotransposons indicates gRNA targeting sites with one mismatch. Arrowhead (green) on the track view indicates poly(A) signal site. (**C**) Representative genomic region comprising LTR12C and other retrotransposons that gained robust expression in HEK293T cells.

Meanwhile, we have shown that dCas9-SunTag-VP64 can induce numerous LTR12C copies; a large number of other LTR families (48% of total activated LTRs) were also expressed after the CRISPRa (Figure 2B). Since recent studies have reported pervasive off-target binding events of dCas9 protein (35,36), we next assessed whether off-target effects of our dCas9-SunTag-VP64 system could be activating these other LTR elements. In accordance with the methods of an earlier study (35), we mapped hemagglutinin-tagged dCas9 (dCas9-HA) binding sites across the genome in HEK293T by performing chromatin immunoprecipitation followed by sequencing (ChIP-seq) with HA antibody. The ChIP-seq peaks called by MACS software (37) showed a substantial enrichment in LTR12C among all the LTRs activated by dCas9-SunTag-VP64, with 99% specificity (Figure 3A). Only a few LTR12E elements (1%) were also detected in the ChIP-seq peaks because the gRNA #15 target sequence was conserved between LTR12C and the activated LTR12E (Supplementary Figure S4). On the other hand, other types of activated LTR families were not enriched with dCas9-HA. Therefore, the expression from other LTR families may result from indirect effects of LTR12C-targeted CRISPRa (Figures 2B and 3B and C). Many of the LTR12C transcripts initiated from the middle of LTR12C elements and subsequently terminated at the following poly(A) signal (Figure 3B). However, we observed transcription elongation events that generated extended transcripts that read through multiple poly(A) signals (Figure 3C). We discovered that >90% of the other upregulated intergenic LTRs (non-LTR12C) were directly downstream of a transactivated LTR12C element and likely a segment of the transcriptional read-through gene isoform (Supplementary Figure S5A and B). Consequently, LTRs or other retrotransposons downstream of LTR12C also showed robust expression, irrespective of dCas9 binding (Figure 3C). Hence, off-target effects of dCas9-SunTag-VP64 targeting LTR12C elements were limited in HEK293T cells.

### VP64 recruitment to *cis-*regulatory elements of LTR12C drives differential H3K27ac and H3K4me3 deposition with different LTR expression patterns

**Figure 4.**
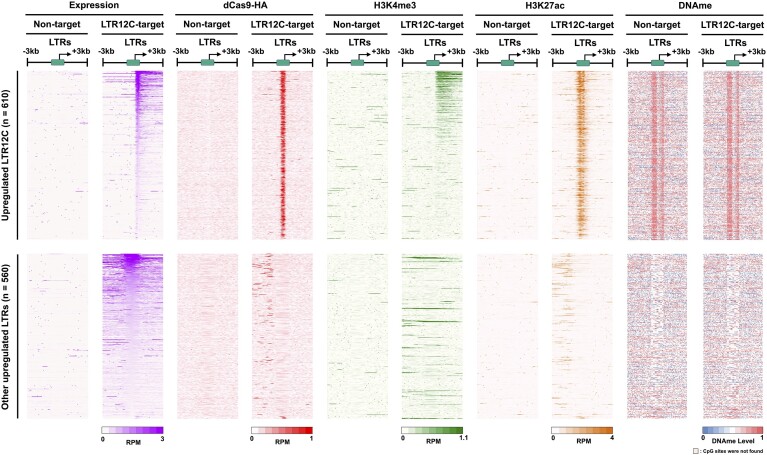
Only a particular group of LTR12Cs acquired both promoter and enhancer activities after CRISPRa in HEK293T cells. Heatmaps for reads per million reads (RPM) were calculated from data of RNA-seq and ChIP-seq using represented antibodies. DNA methylation levels were based on whole genome bisulfite sequencing. The upregulated LTR12C group (upper panel) and the upregulated other LTRs (lower panel) correspond to those in Figure 2B, left panel. The sequencing reads were mapped for the region around LTRs and stacked in order of RPM value for RNA-seq associated with LTRs.

To define the epigenetic mechanism driving LTR12C transactivation in detail, we examined the accumulation of H3K4me3, H3K27ac and DNA methylation levels around TSSs of LTR elements. It is well established that H3K4me3 is highly enriched in active promoters, while H3K27ac is enriched in both potential promoters and enhancers (38,39). The transactivation of LTR12C is highly correlated with binding of dCas9-HA and enrichment of H3K27ac at upstream regions of the TSSs (Figure 4). On the other hand, broad H3K4me3 deposition was observed at downstream regions of the TSSs of extended LTR12C transcripts (Figure 4). Surprisingly, only 20% of transactivated LTR12Cs were marked by both H3K27ac and H3K4me3, and these co-marked LTR12C elements often gave rise to very long transcriptional read-through transcripts (Figure 4 and Supplementary Table S1).

While DNA methylation levels surrounding the LTR12C TSS were heterogeneous at basal state (Figure 4), we did not detect any DNA methylation changes in the transactivated LTR12C elements after CRISPRa modulation. This suggests that the mechanism responsible for the transactivation of LTR12C might be through modulating histone modifications (Figure 4 and Supplementary Figure S6). In addition, we did not identify enrichment of dCas9-HA binding, H3K4me3 and H3K27ac marks at other LTRs that were upregulated after CRISPRa (bottom panel of Figure 4). These results further support the idea that the expression of other LTRs is a consequence of extended LTR12C transcripts (Figures 3C and 4). Taken together, these results highlight the utility of CRISPRa systems to dissect LTR biology and discover differential epigenetic patterns—single-marked (H3K27ac) or co-marked (H3K4me3 and H3K27ac)—that define transactivated LTR12C.

### H3K4me3-marked promoter activity of LTR12C is linked to the downstream internal ERV9 sequences

**Figure 5.**
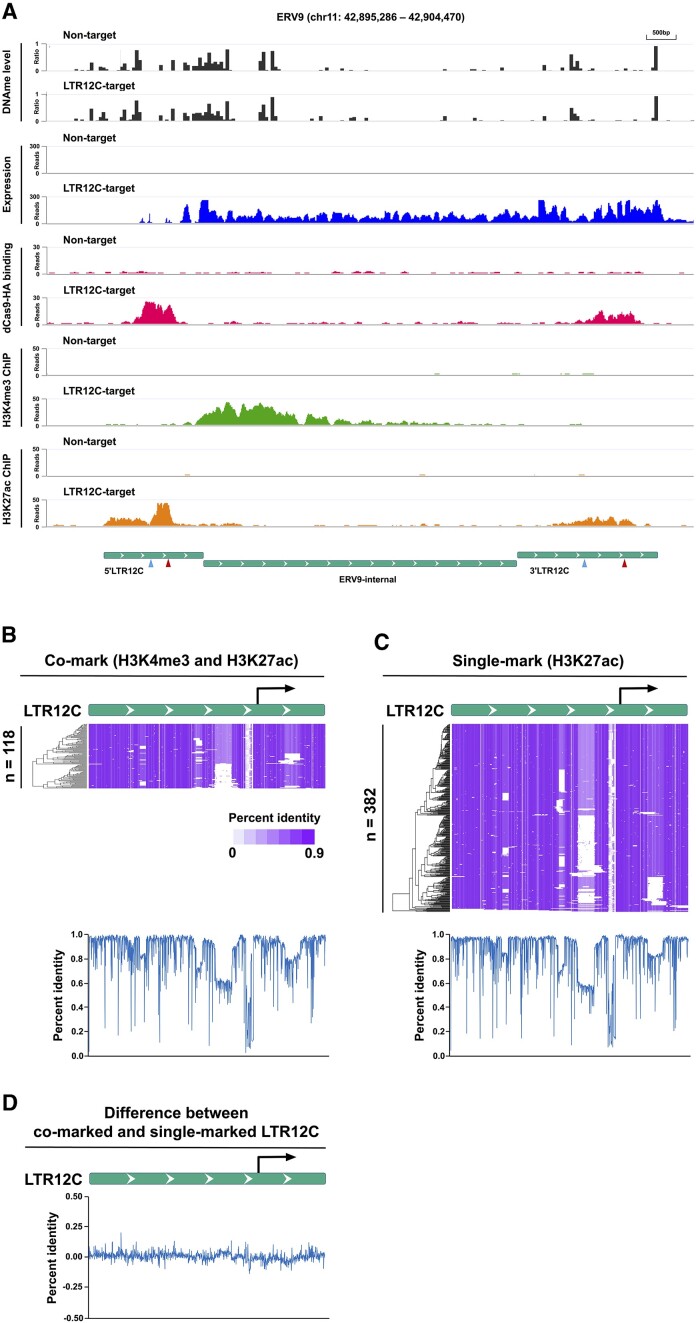
LTR12C located at the 5′ end of putative intact ERV9, but not at the 3′ end, shows H3K4me3-marked promoter activities. (**A**) The track view represents transactivation of LTR12C at a putative intact ERV9 locus in HEK293T cells. Green (bottom) bars indicate the genomic position of LTR12C and the internal region in the putative intact ERV9 with gRNA targeting sites with no (red arrowhead) or one mismatch (blue arrowhead). (**B**) A sequence alignment of LTR12C elements enriched with H3K4me3 and H3K27ac. Each row represents one LTR12C element. Heatmap indicates percent identity (0–90%); blank indicates gaps (upper). The percent identities were also represented by the blue line (lower). (**C**) A sequence alignment of LTR12C elements enriched with only H3K27ac. (**D**) The difference between panels (B) and (C).

LTR12C copies are derived from the ancestral LTR region of ERV9, which infected and propagated rapidly during primate evolution (40). This resulted in the colonization of >2000 copies of LTR12C in the human genome. While majority of LTR12C copies are now solitary LTRs that lost internal ERV9 sequences through recombination events, there still are numerous LTR12C-ERV9 elements present in the human genome. How the presence of the ERV9 sequences impacts the epigenetic regulation of proximal LTR12Cs is unclear in the context of transcription reactivation. Therefore, we first focused on the epigenetic signatures that define the putative intact ERV9 loci. We detected robust transcriptional upregulation of ERV9 loci following LTR12C-targeting CRISPRa (Figure 5A). Whereas H3K27ac was found to be enriched at both 5′LTR12C and 3′LTR12C, only the 5′LTR12C was marked by broad H3K4me3 deposition, which initiated and extended downstream of the TSS or VP64 binding site with limited overlap with H3K27ac peaks (Figure 5A). This atypical signature was consistently detected among most of the transactivated ERV9s (Supplementary Figure S7).

While the 5′LTR and 3′LTR of the retrovirus genome are essentially identical in DNA sequence (41), there is limited knowledge about LTR12C sequence conservation in this context. Therefore, we hypothesized that differences in DNA sequence between co-marked (H3K4me3 and H3K27ac) and single-marked (H3K27ac) LTR12C elements might be linked to their distinct *cis-*regulatory element activities. To assess that, we calculated the percent identity, which is a score for quantitative measurement of similarity between consensus sequence and each copy of LTR12C, for both co-marked and single-marked LTR12C elements (Figure 5B and C). However, there was not a prominent difference between the two groups (Figure 5D). Thus, the distinct *cis-*regulatory element activity of LTR12C was not likely due to the global sequence divergence in evolutionarily conserved regions of LTR12C.

### Co-marked LTR12C epigenetic signatures were also detected after epigenetic therapy

**Figure 6.**
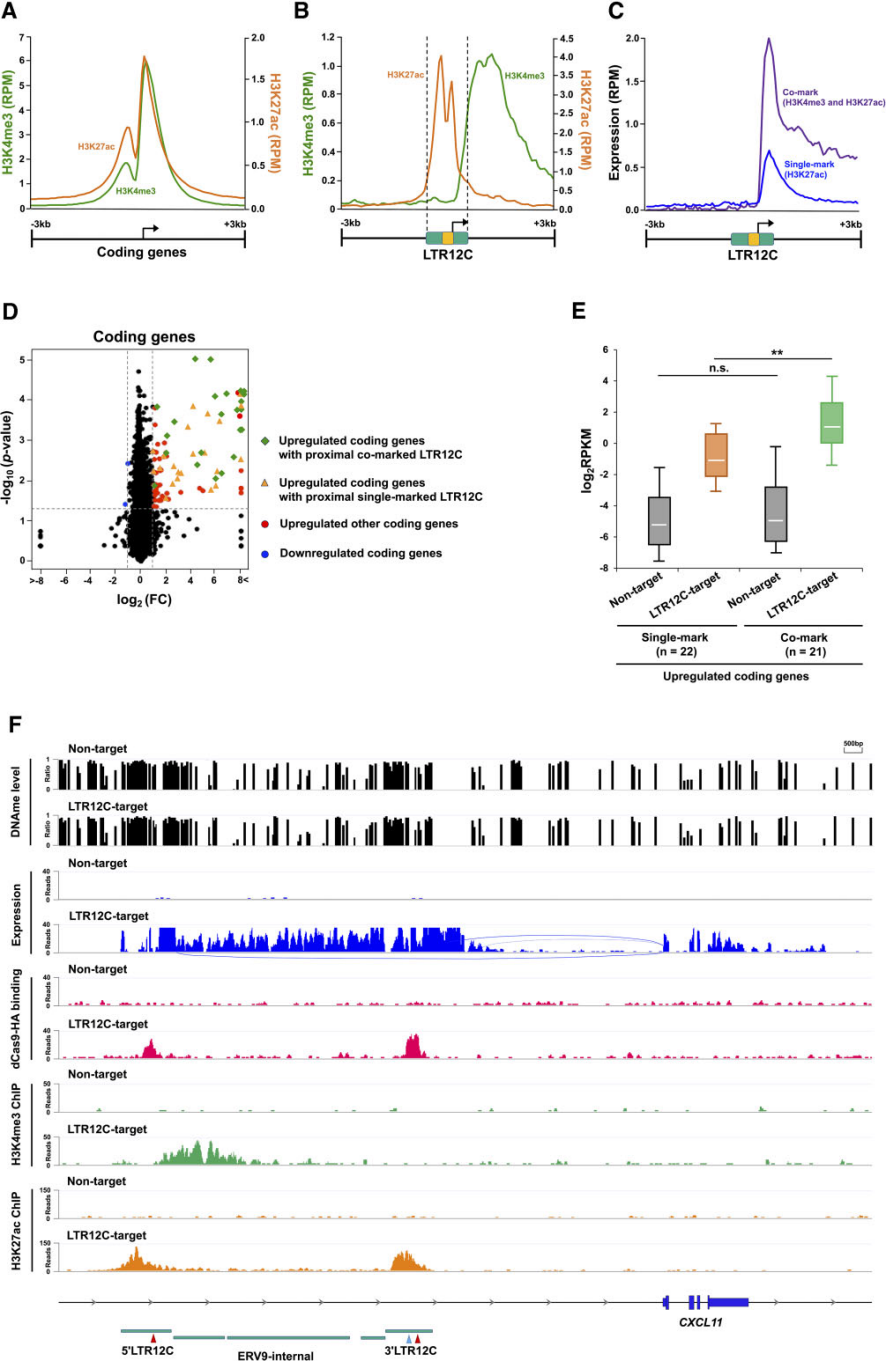
The H3K4me3-marked promoter activities found at downstream sites of LTR12C elements have an impact on expression of downstream host genes in HEK293T cells. (**A**) Occupancy plots showing the average RPM of H3K4me3 (green) and H3K27ac (brown) centered around TSS of host-coding genes. (**B**) LTR12C elements with represented histone modifications after CRISPRa. Yellow (middle) band represents LTR12C’s promoter and enhancer region. (**C**) The average RPM of RNA-seq centered around LTR12C elements enriched with co-mark (H3K4me3 and H3K27ac) or single mark (H3K27ac). (**D**) The volcano plot shows expression changes of coding genes after transactivation of LTR12C. The log_2_FC values and −log_10_(*P*-value) were calculated by comparison with expression of coding genes in cells transfected with LTR12C-targeting gRNA versus cells transfected with nontargeting gRNA. Dashed lines are the threshold of the two-tailed Wilcoxon signed-rank test *P*-value <0.05 (horizontal) or FC > 2 (vertical). (**E**) The distribution of expression level (RPKM) of upregulated coding genes in the represented groups. Expression levels of upregulated coding genes with proximal transactivated H3K27ac-positive LTR12C and co-marked LTR12C are shown by brown box and green box, respectively. Each box represents the data between the 25th and 75th quartiles. The whiskers are drawn down to the 10th percentile and up to the 90th percentile. White bars indicate median values. Adjusted *P*-values were calculated using Mann–Whitney *U* test and Holm’s method: **P < 0.01. (**F**) Representative genomic region showing transactivation of LTR12C and a downstream coding gene (*CXCL11*: C–X–C motif chemokine ligand 11). Green (bottom) bars indicate genomic position of LTR12C and ERV9-internal region with gRNA targeting sites with no (red arrowhead) or one mismatch (blue arrowhead).

We next hypothesized that the distinct *cis-*regulatory element activity of LTR12C might be ascribed to differences in neighbor regions of LTR12C, because many co-marked LTR12C elements had a peak H3K4me3 signal that extended past the LTR12C element and into the ERV9 internal region (Figure 5A and Supplementary Figure S7). We therefore performed comparative analyses examining the peak sites of H3K4me3 and H3K27ac signals around TSS of coding genes and transactivated LTR12C. While both signals largely overlapped with each other at the TSS of coding genes (Figure 6A), the overlap was not seen at the TSS in LTR12C due to a shift of H3K4me3 to downstream regions (Figure 6B). To verify that this signature was not an artifact of CRISPRa technology, we reanalyzed published ChIP-seq data in H1299 lung cancer cells treated with 5-aza-CdR and HDACi combination (8). Indeed, 43% of the transactivated LTR12Cs detected in HEK293T after CRISPRa induction were also activated by epigenetic therapy in H1299 cells (Supplementary Figure S8A). We confirmed that a subset of epigenetic therapy-induced LTR12Cs in H1299 cells also share the unique H3K4me3 and H3K27ac signatures detected in co-marked LTR12Cs in HEK293T cells (Supplementary Figure S8B and C). Furthermore, we queried how the dynamics of bona-fide promoter marks (H3K9ac) or repressive histone marks (H3K9me3, H3K27me3) tracked with co-marked LTR12C transactivation after epigenetic therapy. As expected, H3K9ac deposition mirrored the combined signatures of H3K4me3 and H3K27ac suggesting that these co-marked LTR12Cs are bona-fide promoter units. Interestingly, we observed the presence of H3K9me3 and H3K27me3 across transactivated LTR12Cs in H1299 cells treated with epigenetic therapy (Supplementary Figure S8B) (8). In particular, we observe H3K27me3 accumulation overlapping H3K4me3-enriched regions in the LTR12Cs. This phenomenon could reflect the cellular heterogeneity in the sample where certain subpopulation of cells undergo an ‘epigenetic switch’ to compensate for transcriptional reactivation that is countered with histone methylation (8,15,18).

Notably, the observed co-marked promoter activities were not solely confined to the intact ERV9 loci. A large number of loci adjacent to LTR12C were also enriched with both H3K4me3 and H3K27ac after CRISPRa (Figures 4). Therefore, we explored whether focal and specific sequence features within the downstream regions of LTR12C can distinguish co-marked or single-marked LTR12C elements. Considering that the H3K4me3 peak is present behind the LTR12C TSS and that not all co-marked LTR12Cs are flanked by ERV9, we screened 500 bp downstream region of LTR12C for transcription factor binding sites (TFBSs) enriched in the co-marked LTR12C, but not in single-marked LTR12C candidates. We prioritized biologically relevant TFBSs by removing candidates of transcription factors that were not expressed (RPKM < 1) in HEK293T cells. We detected known TFBSs, such as NFY and GATA2 motifs (8,19,20,34,42), that are important for LTR12C promoter function (Supplementary Figure S9). However, further work must be done to verify the necessity of these enriched TFBS candidates for H3K4me3 deposition in co-marked LTR12Cs.

### H3K4me3- and H3K27ac-marked LTR12C elements robustly activate downstream coding genes

The co-marked LTR12C elements were characterized by higher expression of read-through RNA transcripts in comparison with single-marked LTR12C (Figures 4 and 6C). The results suggest that the co-marked LTR12C elements can serve as alternative promoters to influence downstream host-coding gene expressions (Figure 6D–F and Supplementary Figure S10A and B). In fact, we identified 21 upregulated coding genes with proximal co-marked LTR12C without changes of H3K4me3/H3K27ac marks at their canonical promoter regions (Figure 6D and E). Although other 22 coding genes with proximal single-marked LTR12C were also upregulated, the expression levels of them were lower than those of 21 coding genes with proximal co-marked LTR12C (Figure 6D and E). Whereas the genes with diverse functions were involved in the list (Supplementary Table S2), importantly several immune response genes, such as *CXCL11* and *CCR4* (C–C motif chemokine receptor 4), were likely upregulated through this mechanism. Since H3K4me3/H3K27ac marks were not enriched at canonical promoter region of *CXCL11*, the upregulation of the gene is likely derived from transactivated LTR12C element (Figure 6F). In fact, some splicing events were detected that coupled LTR12C to the first exon of *CXCL11* gene (Figure 6F). These results are consistent with a previous report showing the activation of interferon response immune genes by an LTR family of MER41-derived promoter (4). Furthermore, 35 coding genes also showed upregulation without proximal transactivated LTR12C (Figure 6D and Supplementary Table S2). Whereas the mechanism of this upregulation was unclear, we reanalyzed publicly available Hi-C data in wild-type HEK293T cells (43), and found that 26 of the upregulated genes including interferon responding genes were located in same topologically associating domain as distal transactivated LTR12C (Supplementary Table S2 and Supplementary Figure S11A–D). This result supports the potential enhancer activity that these transactivated LTR12Cs could have on the host genes, but warrants further work. Here, we show evidence for the co-option of LTR12C elements with host immune response through potential alternative promoter or enhancer functions.

## Discussion

It is known that epigenetic inhibitors induce potent transcriptional competence of LTR12C. However, the specific roles of LTR12C in the host transcriptional regulatory network have not been characterized yet due to the inadvertent global alteration of gene expression caused by epigenetic inhibitor treatment. Here, we undertook a functional dissection of LTR12C using CRISPRa technology to decouple the consequences of LTR12C activation from global epigenetic changes induced by epigenetic inhibitors. Since a single gRNA-based conventional CRISPRa tool was often insufficient for robust gene activation, multiple gRNAs have been simultaneously designed at a promoter region (44,45). Here, we leveraged the repetitive and conserved sequence nature of LTRs to design an efficient single gRNA targeting multiple copies of LTR12C. Moreover, our CRISPRa tool consists of SunTags separated by 22-amino-acid linkers, which should improve the transactivation capacity of the CRISPRa technology. Our method allowed the transactivation of >600 copies of LTR12C using the dCas9-SunTag-VP64, in contrast to only ∼100 copies activated by a DNA methylation inhibitor. More importantly, when using our single gRNA-based CRISPRa system, we did not detect the pervasive off-target effects in mammalian cells that have been published by other groups (35,36). Lastly, our data indicate that the reactivation of LTR12C using dCas9-SunTag-VP64 is independent of the DNA methylation status in these elements. Therefore, the dCas9-SunTag-VP64 system provides better representation of the robust transactivation potential of transcriptionally competent LTR12C copies.

While the majority of LTR12C elements gained H3K27ac-marked enhancer activities, only 20% of them showed H3K4me3-marked promoter activities after CRISPRa. We observed a shift in the H3K4me3 peak toward the downstream TSS region in transactivated 5′LTR12C copies, which coincides with the presence of downstream internal region of ERV9 sequences. Further, we confirmed this atypical shift of the H3K4me3 peak to downstream regions in eight 5′LTR12C copies with putative intact ERV9 and two solitary LTR12C loci by reanalyzing data from a previously published study (8) (Supplementary Figure S8B and C). It is important to note that several solitary LTR12C elements without nearby ERV9 sequences also gained H3K4me3 peaks (Figure 4 and Supplementary Figure S8C). One potential explanation could be that additional transcription factor binding events after the TSS in the solitary LTR12C elements could lead to broad H3K4me3 deposition. Further functional studies are necessary to elucidate the causal relationship between the particular regions adjacent to LTR12C and promoter activity.

In comparison to a previous report (24), the superiority of dCas9-SunTag-p300 in transactivation of *cis-*regulatory elements was not observed in the case of LTR12C. This discrepancy may be due to the distinct transactivation machinery of dCas9-SunTag-VP64 and dCas9-SunTag-p300. VP64 directly facilitates recruitment of the transcription complex, including RNA polymerase II, to the intended *cis-*regulatory element (46). On the other hand, p300 is known to interact with hundreds of proteins, but is most appreciated for its ability to add acetyl groups to various lysines on histone tails, particularly H3K27ac (47,48). Indeed, H3K27ac enrichment levels associated with high expression of dCas9-SunTag-p300-targeted LTR12C were significantly higher than those at control LTR12C, but only a modest trend was observed in dCas9-SunTag-VP64-targeted LTR12C (Supplementary Figure S2B). While the exact molecular mechanisms involved require further elucidation, our results indicate that the previously reported superiority of dCas9-p300 in activating gene expression does not hold true for LTR12C and potentially other noncanonical promoters. This underscores the complexity of the activation of *cis-*regulatory elements, which requires a nuanced approach for further understanding the diverse mechanisms at play.

Recent comprehensive analyses using deep sequencing technologies have demonstrated that some parts of LTR elements have been pervasively co-opted as *cis-*regulatory elements for host-coding genes (3–5). Here, we showed that CRISPRa-induced LTR12C promoters show epigenetic signatures distinct from those of canonical gene promoters. The transactivated LTR12C elements could be categorized into two groups composed of co-marked (H3K4me3 and H3K27ac) and single-marked (H3K27ac) LTR12C elements. The induced expression level of co-marked LTR12C elements was itself substantially higher than that of single-marked LTR12C elements. Furthermore, the co-marked LTR12C elements retained their high expression capacity over the downstream regions to create run-through transcripts. These LTR12C copies elicited strong transcriptional responses in downstream coding genes, including some immune-related genes, suggesting the functional significance of LTR12C in regulating key immune response pathways. These functional consequences of histone H3K4me3 modification in the extension of transcripts were in line with a recent report showing a novel role of H3K4me3 in transcriptional regulation (49). While H3K4me3 has been proposed to regulate initiation of transcription, Wang *et al.* demonstrated a distinct role for H3K4me3 in transcriptional elongation by releasing paused RNA polymerase II (49). Further, the broad peaks of H3K4me3 have been associated with enhancer features by earlier studies (50–52). Therefore, the regulation of host-coding genes by LTR12C was largely brought about by the co-marked LTR12C elements.

This work highlights the utility of the dCas9-SunTag-VP64 tool to modulate specific LTR subfamilies to dissect the *cis-*regulatory contribution to host gene regulation and provide a potential therapeutic tool to reactivation of the immune response.

## Supplementary Material

gkae498_Supplemental_Files

## Data Availability

The raw and processed datasets from this study have been submitted to the NCBI GEO (http://www.ncbi.nlm.nih.gov/geo/) under accession number GSE243551. Source code was deposited in Figshare (doi: 10.6084/m9.figshare.24906660).
