# Supplementary material for: Efficient activation of hundreds of LTR12C elements reveals cis-regulatory function determined by distinct epigenetic mechanisms
Source: Nucleic Acids Res. 2024 Jun 14;52(14):8205–17. doi: 10.1093/nar/gkae498 (PMC11317136; doi:10.1093/nar/gkae498)
Supplement: gkae498_Supplemental_Files [file gkae498_supplemental_files.zip › Supplementary_Figures.pdf]

Supplementary Figure.S1

5’tgtgagaggtgacagcgtgctggcagtcctcacagccctcgctcgctctcggcgcctcctctgcctgggct

#1 #2

cccactttggcggcacttgaggagcccttcagcccgccgctgcactgtgggagcccctttctgggctggccaa

ggccggagccggctccctcagcttgcagggaggtgtggagggagaggcgcgggcgggaaccggggctgcgcgc

#3

ggcgcttgcgggccagctggagttccgggtgggcgtgggcttggcgggccccgcactcggagcggccggccgg

#4 SP1

cccgccggcccgggcagtgaggggcttagcacccgggccagcggctgcggagggtgtactgggtcccccagc

#5

agtgccggcccaccggcgctgcgctcgatttctcgccgggccttagctgccttcccgcggggcagggctcggg

acctgcagcccgccatgcctgagcctcccccccccctccgtgggctcctgtgcggcccgagcctccccgacga

#6

gcgccgcccccctgctccacggcgcccagtcccatcgaccacccaagggctgaggagtgcgggcgcacggcgcg

#7

ggactggcaggcagctccacctgcagccccggtgcgggatccactgggtgaagccagctgggctcctgagtct

#8

ggtggggacttggagaacctttatgtctagctaagggattgtaaatacaccaatcggcactctgtatctagct

#9 NFY

caaggtttgtaaacacaccaatcagcaccctgtgtctagctcagggtttgtgaatgcaccaatcgacactctg

#10 #11 #12

ttatctagctactctggtggggacttggagaacctttatgtctagctcagggattgtaaatacaccaatcggc

actctgtatctagctcaaggtttgtaaacacaccaatcagcaccctgtgtctagctcagggtttgtgaatgca

#13

ccaatcgacactctgtatctagctaatctggtggggacgtggagaacctttgtgtctagctcagggattgtaa

#14 #15

acgcaccaatcagcaccctgtcaaaacggaccaatcagctctctgtaaaacagaccaatcggctctctgtaaa

#16 #17 #18

atggaccaatcagcaggatgtgggtgggccagataagagaataaaagcaggctgcccgagccagcagtggca

#19 #20 GATA2 #21

acccgctcgggtccccttccacactgtggaagctttgttctttcgctctttgcaataaatcttgctgctgctc

actctttgggtccacactgcctttatgagctgtaacactcaccgcgaaggtctgcagcttcactcctgaagcc

agcgagaccacgagcccaccgggaggaacgaacaactccagacgcgccgccttaagagctgtaacactcaccg

cgaaggtctgcagcttcactcctgagccagcgagaccacgaacccaccagaaggaagaaactccgaacacatc

cgaacatcagaaggaacaaactccggacgcgccgcctttaagagctgtaacactcaccgcgagggtccgcggc

ttcattcttgaagtcagtgagaccaagaacccaccaattccggacaca 3'

Supplementary Figure S1. The positions of gRNAs and transcription factor binding sites on consensus sequence of LTR12C elements, related to Figure 1C.

The consensus sequence of LTR12C elements was obtained from Dfam database (https://dfam.org). The positions of gRNAs are represented by underline. Known transcription factor binding sites are represented by each color (8)(32). Arrow indicates presumptive TSS.

Supplementary Figure.S2

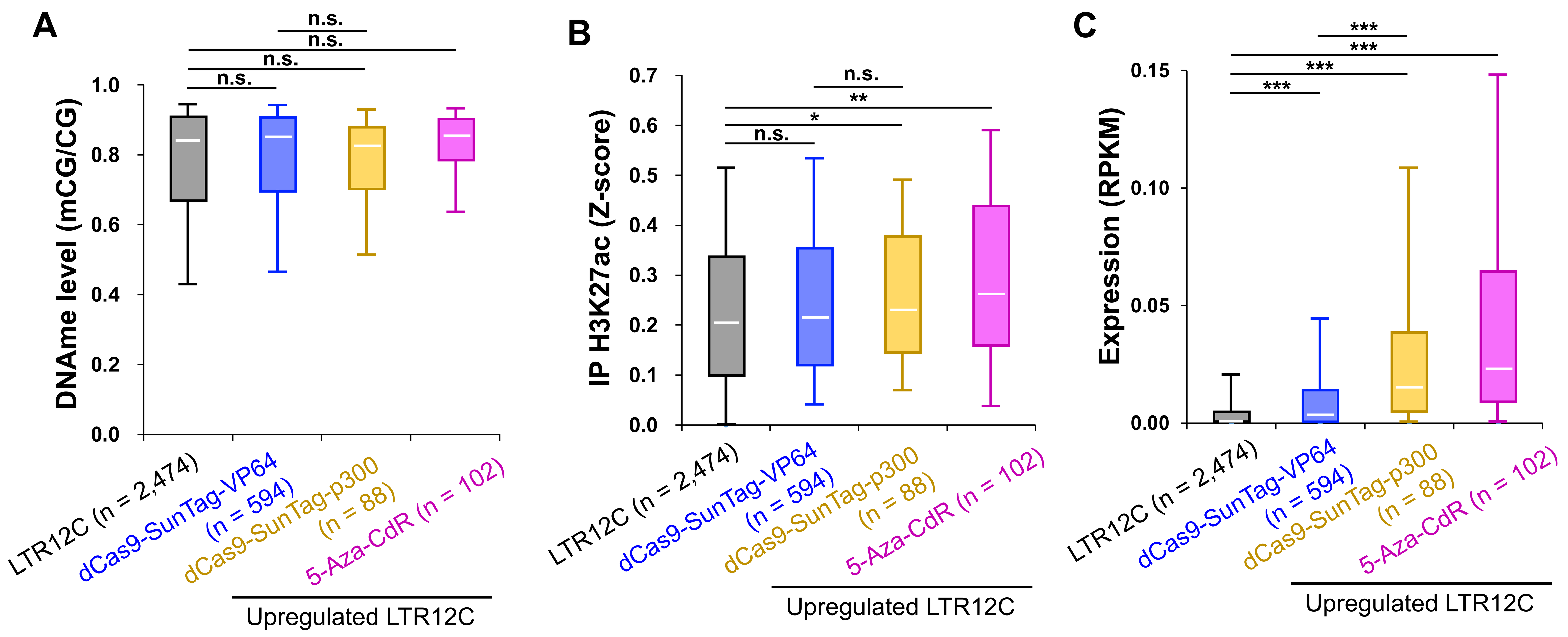

Supplementary Figure S2. dCas9-Suntag-VP64 has a lower target preference for LTR12C copies than dCas9-Suntag-p300 and 5-Aza-CdR

(A) The distribution of basal DNA methylation (DNAme) levels of LTR12C in the represented groups in HEK293T cells. The number of analyzed LTR12C copies in each group is decreased from 610 to 594, from 90 to 88, and from 109 to 102, respectively, due to data availability. "LTR12C (n = 2,474)" refers to LTR12C copies that were deposited in RepeatMasker. Each box represents the data between the 25th and 75th quartiles. The whiskers are drawn down to the 10th percentile and up to the 90th percentile. White bars indicate median values. *P*-values were calculated using Mann-Whitney U test: (∗) P < 0.05, (∗∗) P < 0.01, (∗∗∗) P < 0.001. (B) The distribution of basal H3K27 acetylation (H3K27ac) levels of LTR12C in the represented groups in HEK293T cells. (C) The distribution of basal RNA expression level of LTR12C in the represented groups in HEK293T cells.

Supplementary Figure.S3

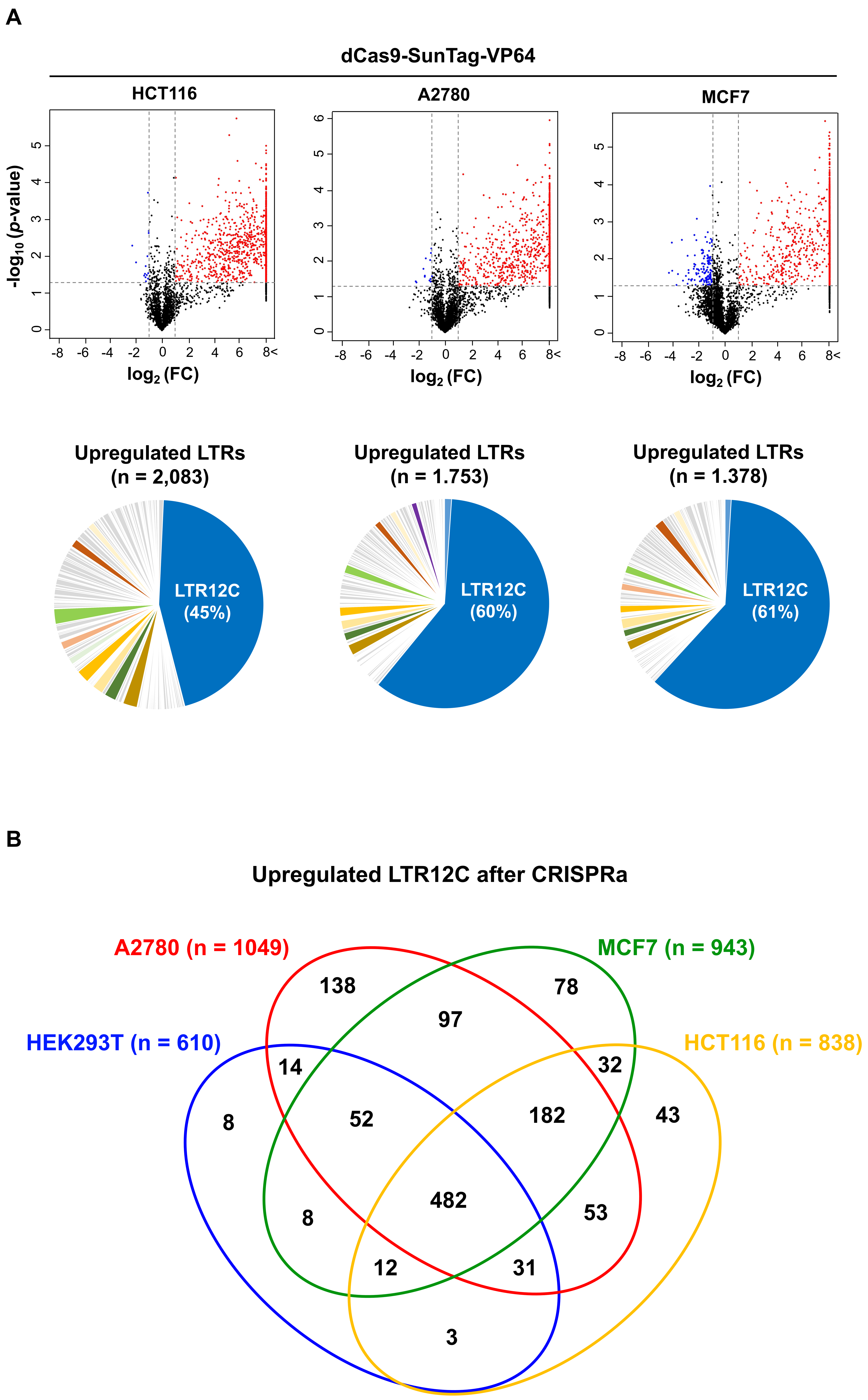

Supplementary Figure S3. The single gRNA-based dCas9-Suntag-VP64 system transactivates a large number of LTR12C copies in three different human cell lines

(A) The volcano plots show expression changes of LTR copies (not only LTR12C) after transfection of dCas9-Suntag-VP64 plasmid construct with LTR12C-targeting gRNA in three cell lines. The $\log_2$FC values and $-\log_{10}$(p-value) were calculated by comparison with expression of LTR copies in cells transfected with constructs expressing non-targeting gRNA. Black dashed lines are the threshold of the two-tailed Wilcoxon Signed-Rank Test P-value < 0.05 (horizontal) or FC > 2 (vertical). The red and blue points represent upregulated and downregulated LTR copies with statistical significance, respectively. The pie charts show percentage of the upregulated LTR copies in each LTR families. The top 10 most highly abundant LTR families are highlighted with distinct colors. (B) Venn diagram shows LTR12C copies overlapping among upregulated LTR12C by dCas9-SunTag-VP64 in each cell line.

Supplementary Figure.S4

**LTR12C (Consensus sequence)**	TCTGGTGGGGACGTGGAGAACCTTTGTGTCTAGCTCAGGGATTGTAAACGCACCAAT
LTR12E (chr4:2,764,081-2,765,138)	TCTGGCGGGGACGTGAAGAACCTTTGTGTCTAGCTCAGGGATTGTAAACACACCAAT
LTR12E (chr4:105,502,770-105,504,245)	CTGGTTGGGGACGTGAAGAACCTTTGTGTCTAGCTCAGGGATTGTAACTACACCAAT
LTR12E (chr5:19,142,174-19,143,443)	TCTGGTGGGGAGGTGGAGAACCTTTATGTCTAGCTCAGGGATTATAACTACACCAAT
LTR12E (chr6:3,027,755-3,028,971)	TGTGGTGGGGACGTGGAGAACCTTTATGTCTAGCTCAGGGATTGTAAACACACCAAT
LTR12E (chr8:84,202,405-84,203,702)	TCTGGTGGGGACATGGAGAACCTTTATGTCTAGCTCAGGGATTGTAAATACACCAAT
LTR12E (chr14:56,306,057-56,307,379)	TCTGGTGGGGACGTGGAGAACCTTTGTATCTAGCTCAGGGATTGTAAACGCACCAAT

dCas9-HA binding LTR12E

Supplementary Figure S4. The gRNA-targeted region in LTR12C is highly conserved in six activated LTR12E copies

The gRNA-targeted region in LTR12C and the corresponding regions in LTR12E copies are shown as underlined. The mismatched sequences in LTR12E copies are represented in gray.

Supplementary Figure.S5

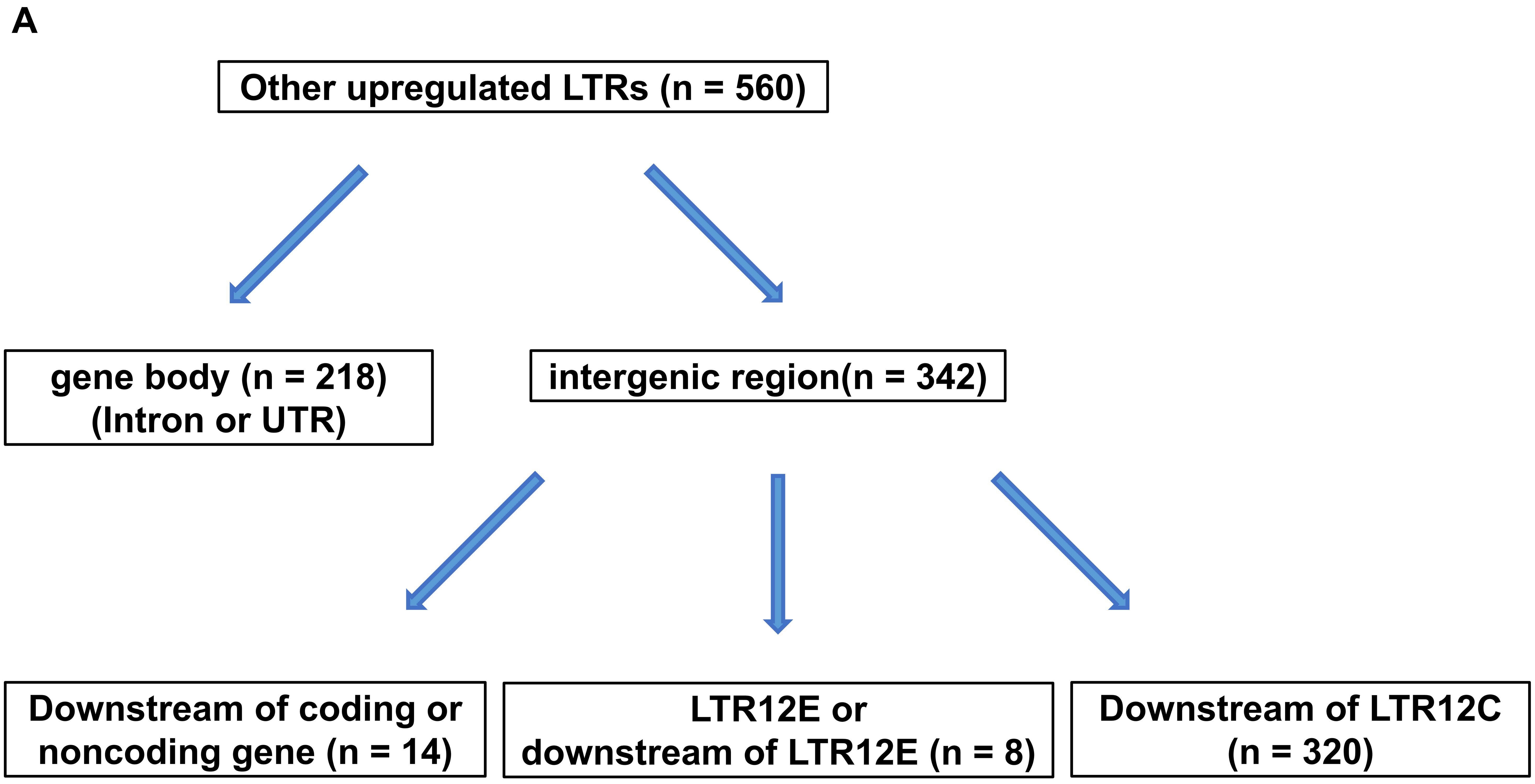

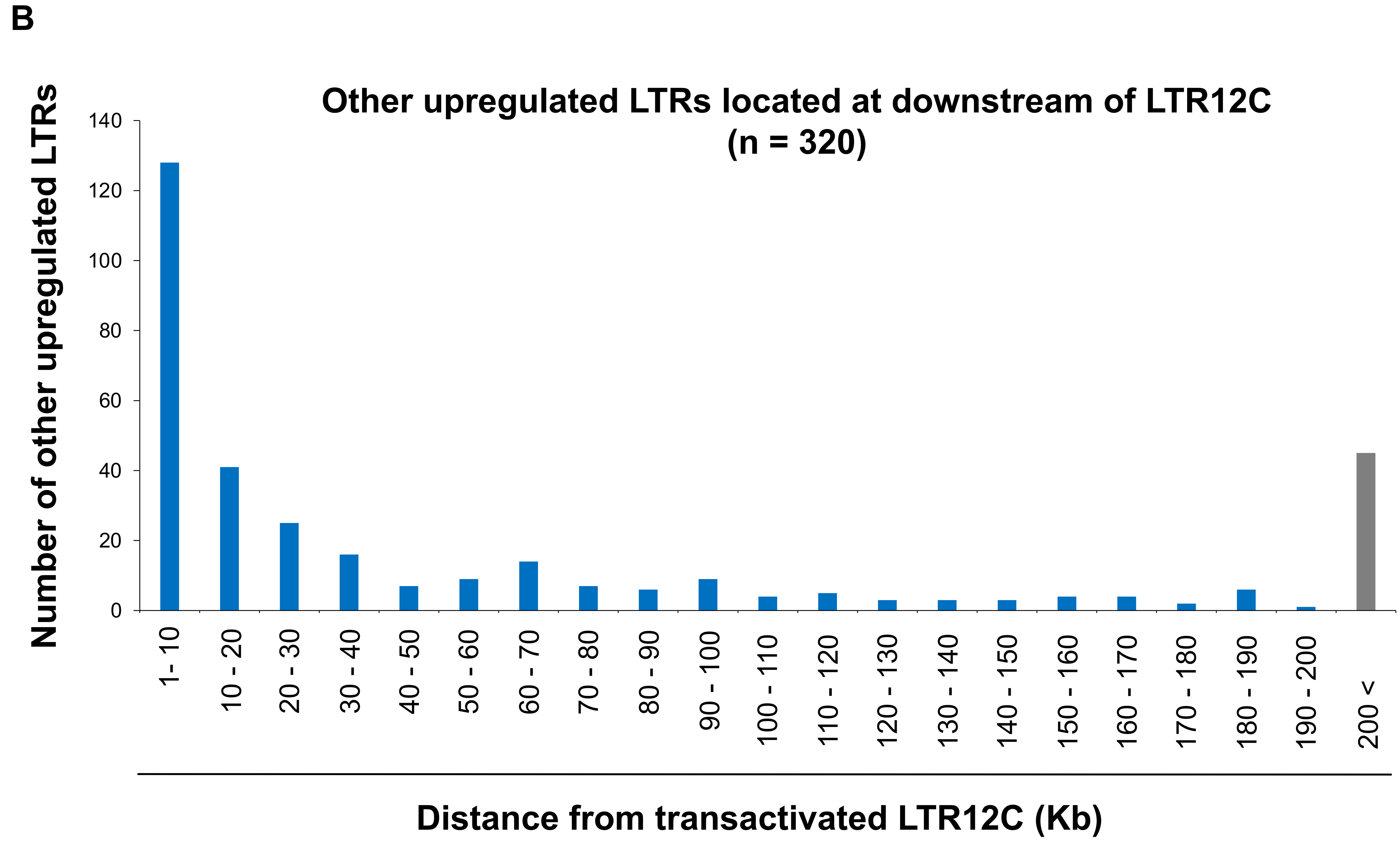

Supplementary Figure S5. Most of the other upregulated intergenic LTRs were located at downstream regions of LTR12C elements, related to Figure 2B.

(A) The other upregulated LTRs overlapped with host gene bodies were excluded to focus on transcripts driven by LTRs. The remaining other upregulated intergenic LTRs were further divided into three groups based on their locations. (B) The bar graph shows distance from transactivated LTR12C to other intergenic LTRs located at downstream of LTR12C.

Supplementary Figure.S6

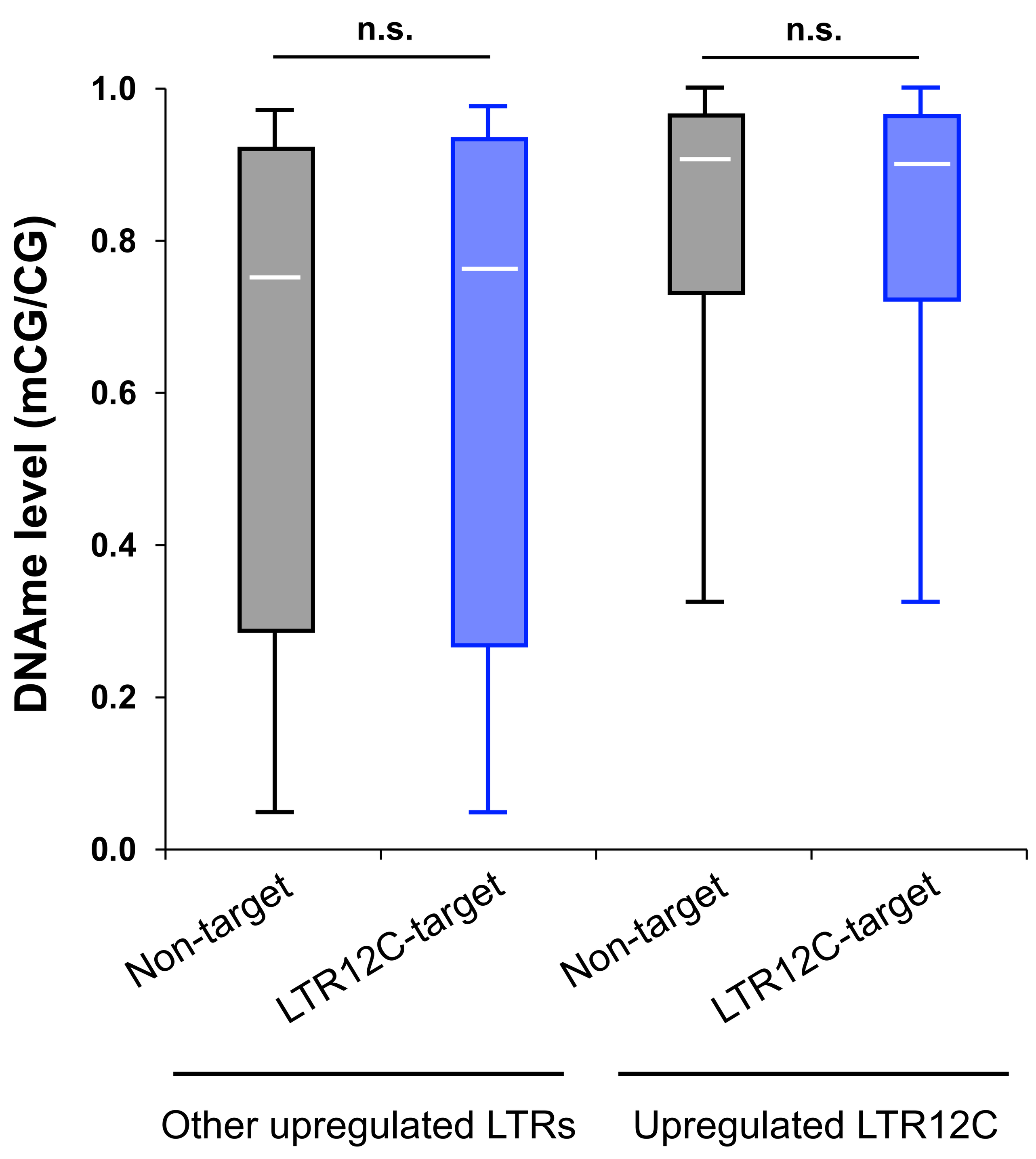

Supplementary Figure S6. DNA methylation levels are not changed by transactivation of LTR12C in HEK293T cells, related to Figure 4.

Each box represents the data between the 25th and 75th quartiles. The whiskers are drawn down to the 10th percentile and up to the 90th percentile. White bars indicate median values. *P*-values were calculated using Mann-Whitney U test.

Supplementary Figure.S7

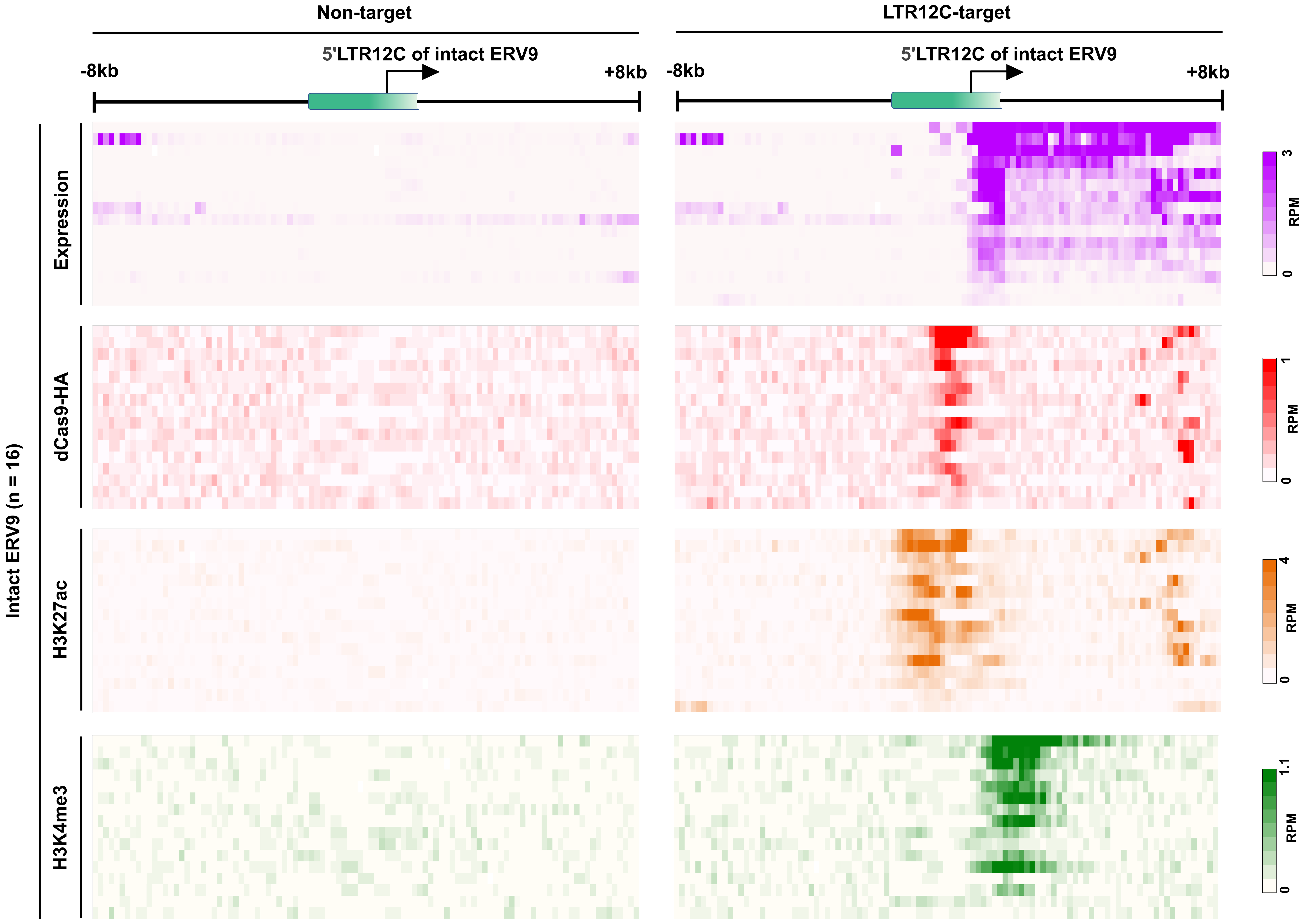

Supplementary Figure S7. Preferential deposition of H3K4me3 at the 5'LTR12C of intact ERV9 elements in HEK293T cells

The RPM values were calculated from RNA-seq data and ChIP-seq using represented antibodies. The sequencing reads were mapped for the region around 5'LTRs of 16 transactivated intact ERV9 elements and stacked in order of RPM value for RNA-seq associated with the 5'LTRs.

Supplementary Figure.S8

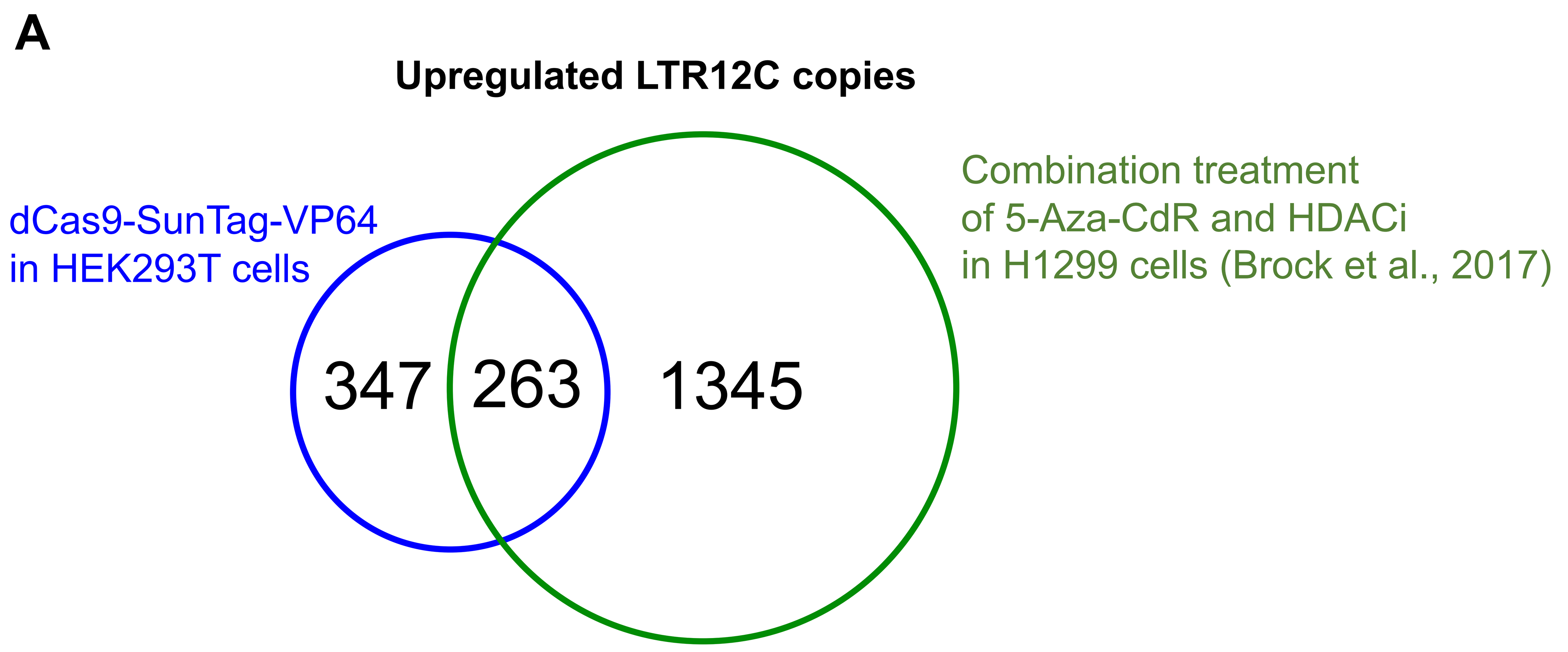

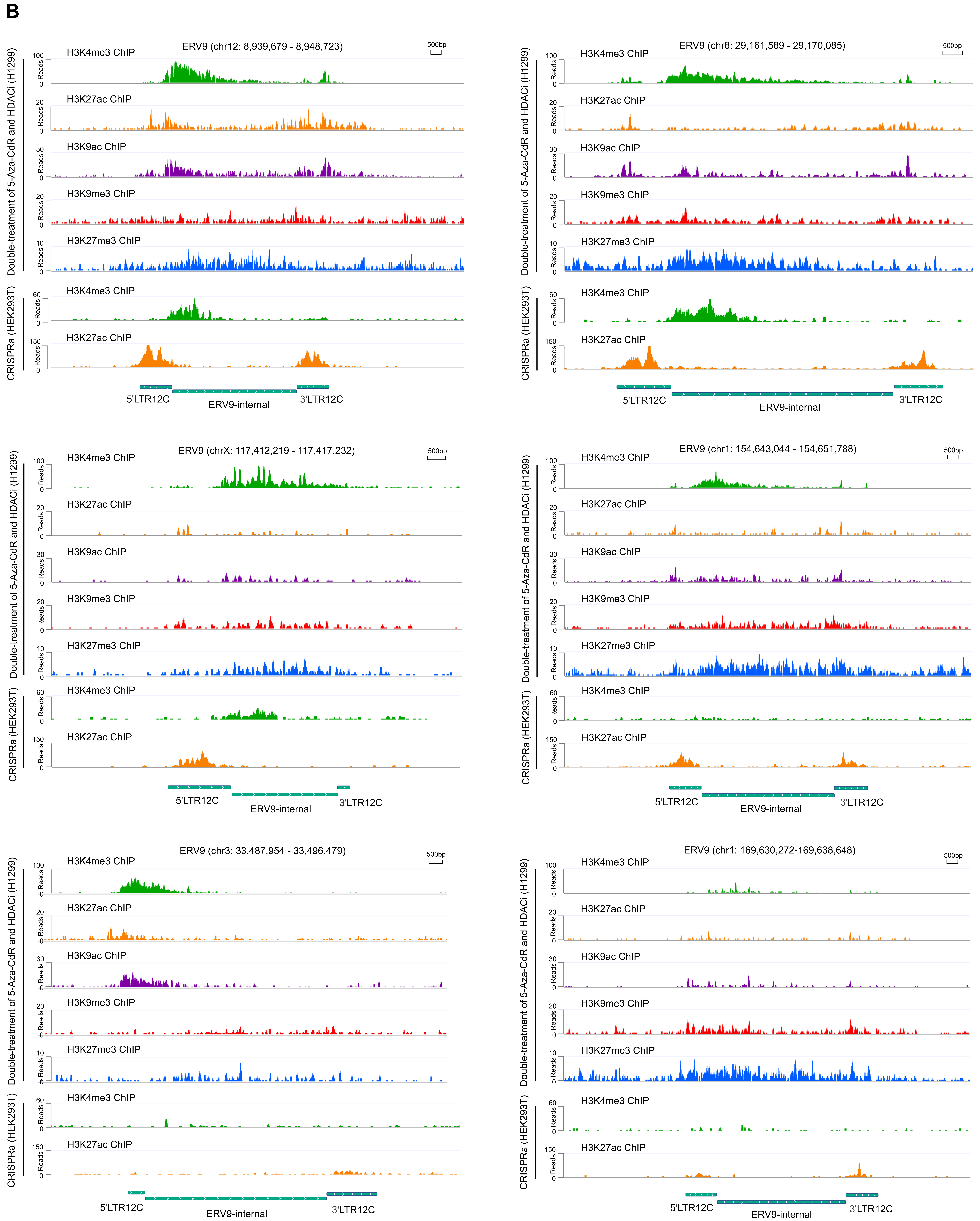

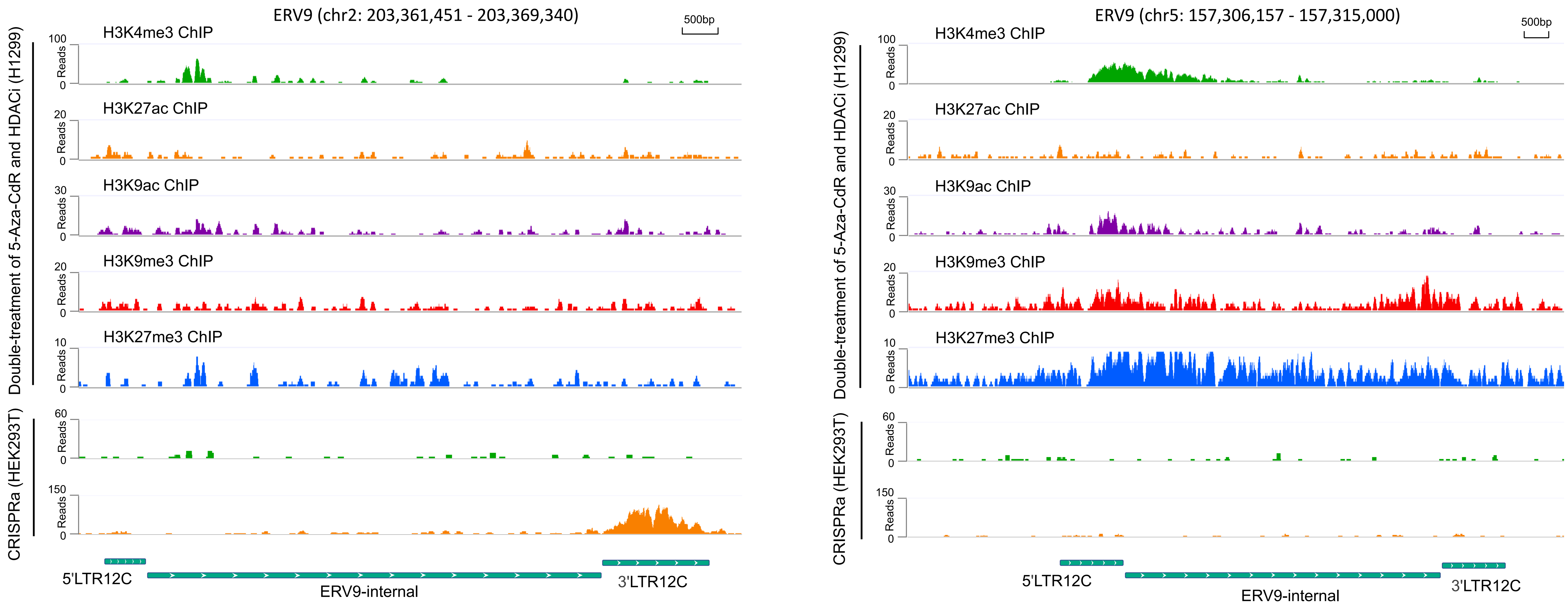

C

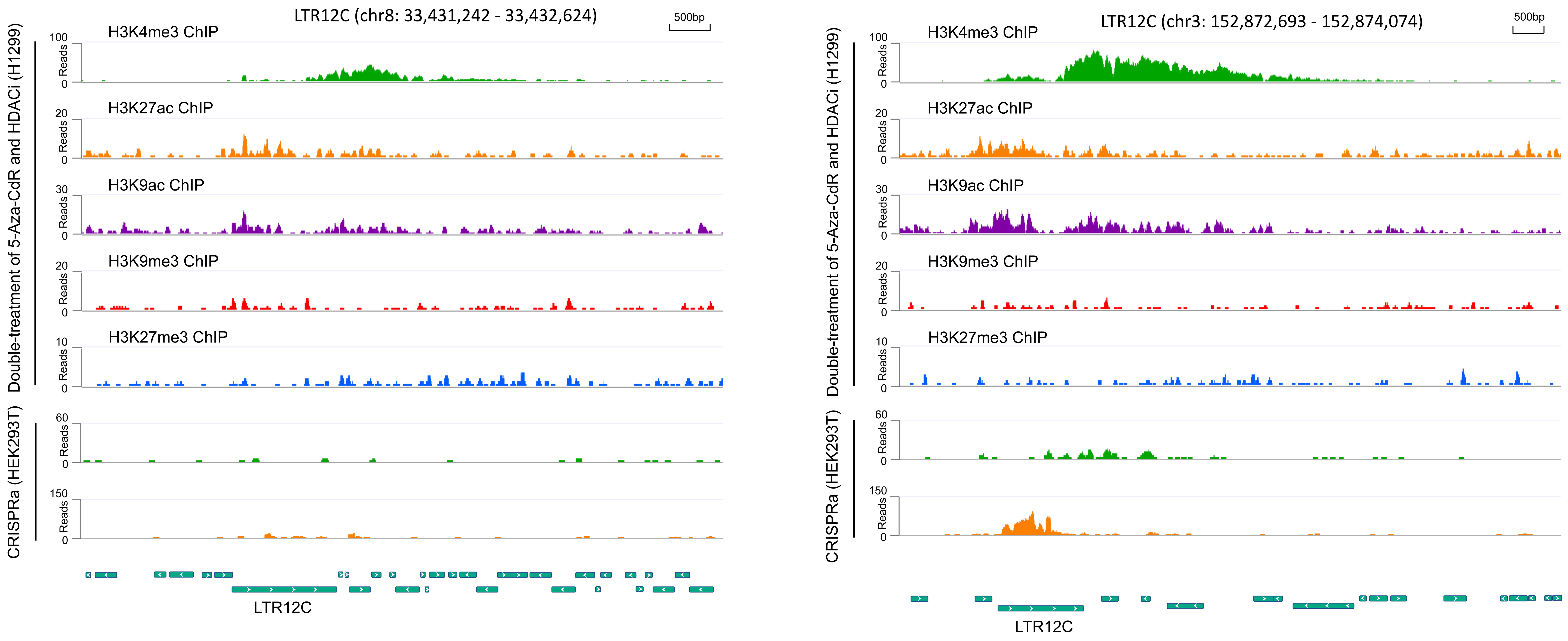

Supplementary Figure S8. The shift of the H3K4me3 peak to downstream regions of LTR12C is confirmed in publicly available data sets after treatments of epigenetic inhibitors in H1299 cells. (A) Venn diagram shows LTR12C copies overlapping between upregulated LTR12C by dCas9-SunTag-VP64 in HEK293T cells and epigenetic inhibitors in H1299 cells. (B) Eight representative putative intact ERV9 and (C) two solitary LTR12C loci are shown. Green bars indicate genomic positions of LTR12C, ERV9-internal region, or other retrotransposons.

Supplementary Figure.S9

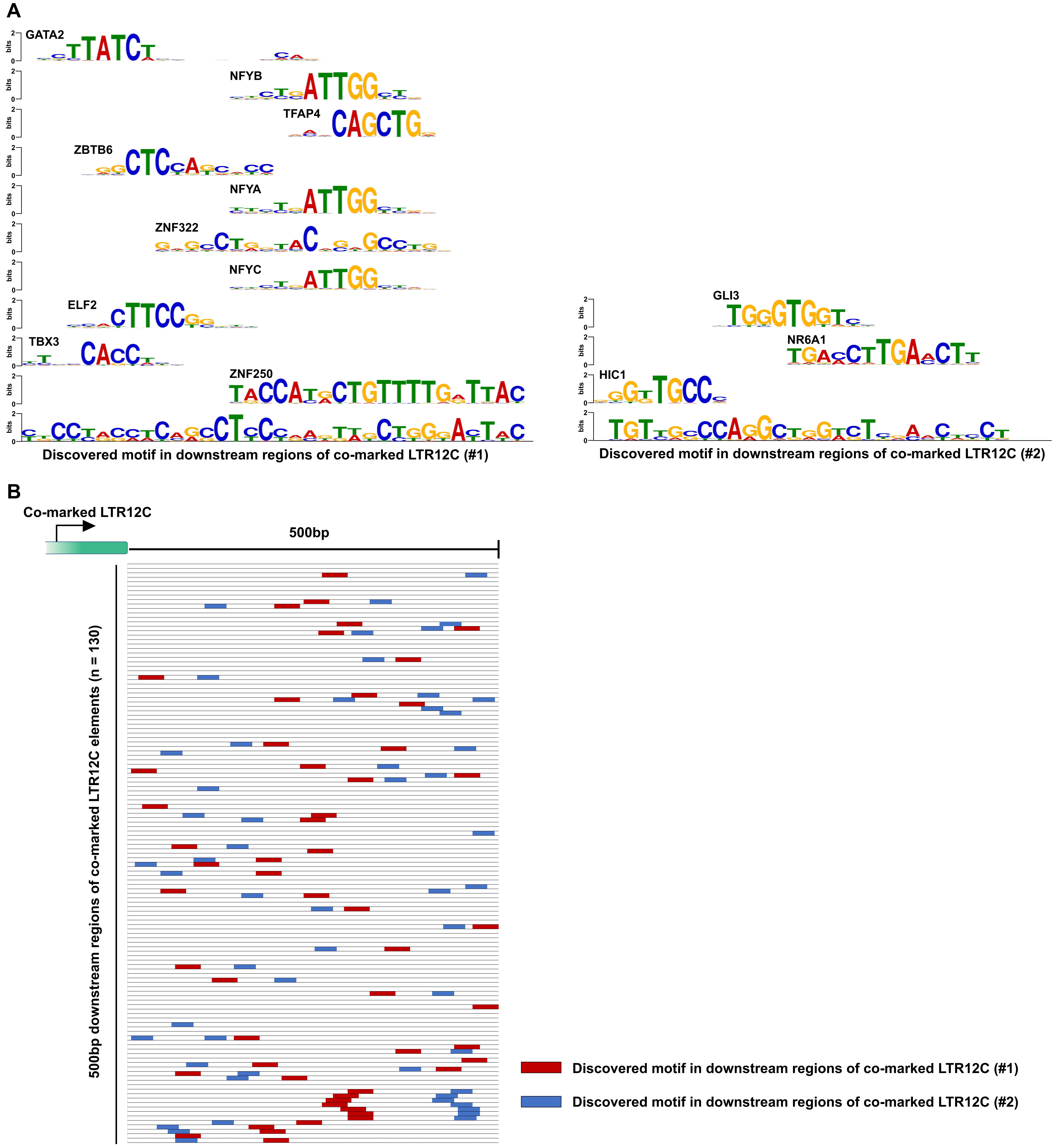

Supplementary Figure S9. Unique transcription factor binding sites discovered in the downstream regions of co-marked LTR12C elements

(A) The logos represent the most significant two motifs that were discovered in the 500bp downstream region of the co-marked LTR12Cs (detected by MEME) and the associated transcription factor binding sites (detected by Tomtom). These transcription factor motifs were derived from highly expressed transcription factors in HEK293T cells (RPKM >1) and verified to be absent in single-marked LTR12Cs. (B) The motif #1 and motif #2 regions were detected in 54 and 55 co-marked LTR12C elements, respectively.

Supplementary Figure.S10

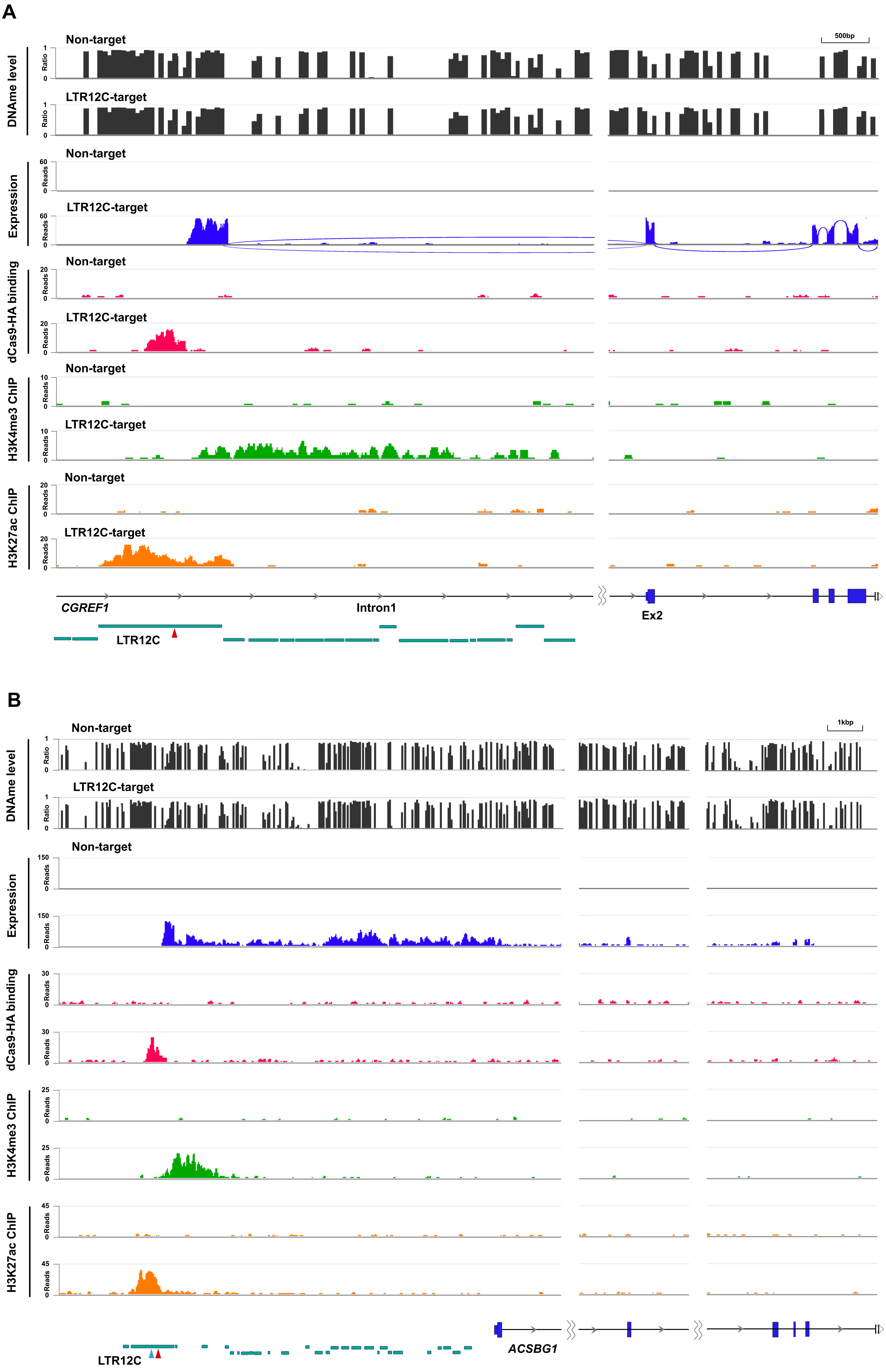

Supplementary Figure S10. Representative genomic regions showing upregulation of host coding genes with proximal co-marked LTR12C elements and their downstream regions.

(A) *CGREF1* (Cell Growth Regulator With EF-Hand Domain) and (B) *ACSBG1* (acyl-CoA synthetase bubblegum family member 1) with proximal co-marked LTR12C elements. Green bars indicate genomic position of LTR12C or other retrotransposons.

Supplementary Figure.S11

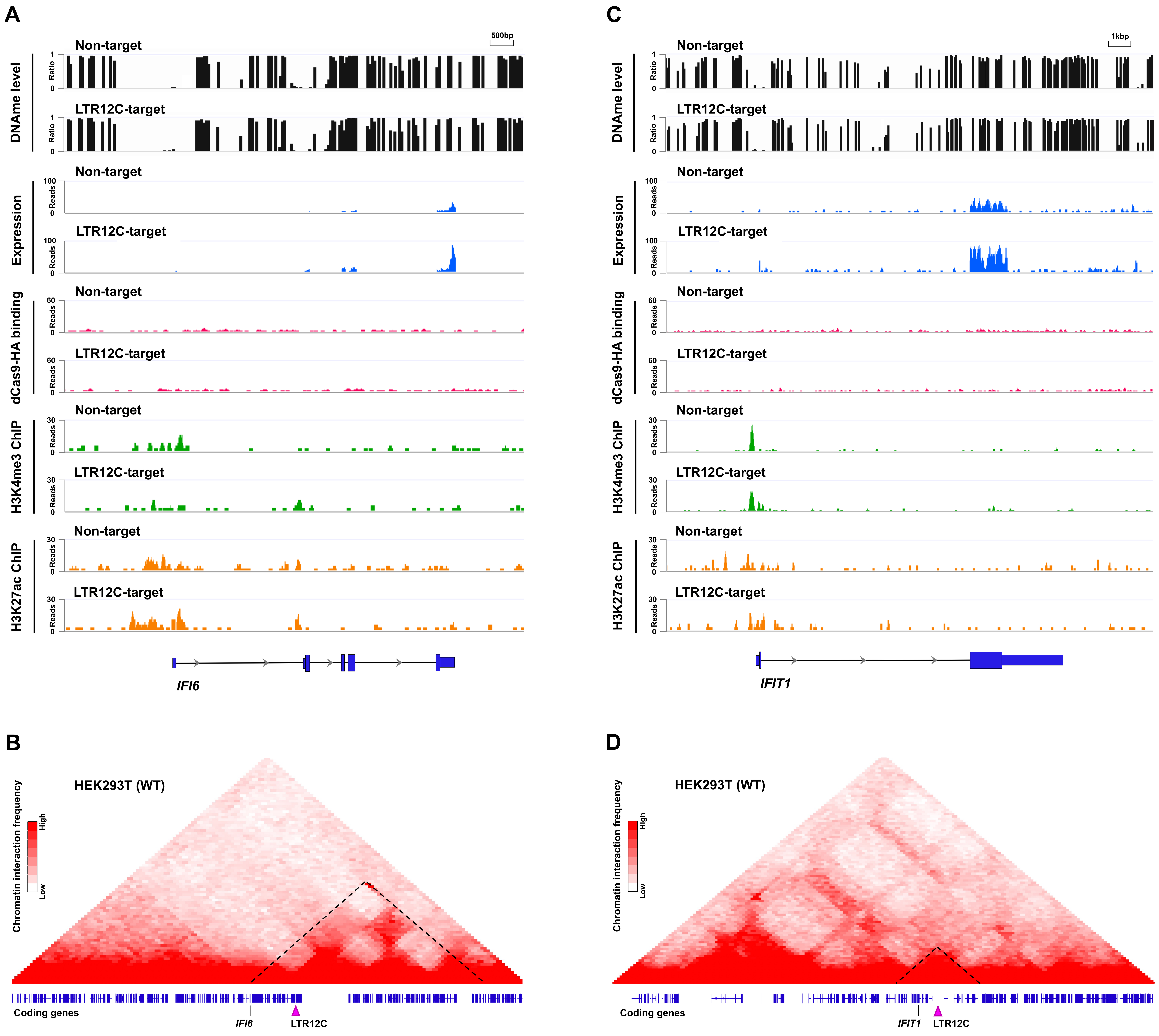

Supplementary Figure S11. The upregulated interferon responding genes were located in same TAD as transactivated LTR12C

The track views represent upregulated immune gene (A) *IFI6* (Interferon alpha inducible protein 6) and (B) *IFIT1* (interferon induced protein with tetratricopeptide repeats 1) without proximal transactivated LTR12C. Chromatin interaction maps were created using Yang's Hi-C data in wild-type HEK293T cells (40). Blue bars indicate genomic positions of coding genes in neighbor region of (C) *IFI6* and (D) *IFIT1*. Arrow heads indicate potions of transactivated LTR12C after CRISPRa. TADs are represented by dotted line.
